# Measuring Indigenous Cultural Strengths: a Systematic Review of a Decade of Approaches

**DOI:** 10.1007/s11121-026-01903-5

**Published:** 2026-03-27

**Authors:** Melissa L. Walls, Danica Love Brown, Victoria M. O’Keefe, Evan J. White, Miigis Gonzalez, Ivy Blackmore, Tara L. Maudrie, Meenakshi Richardson, Timothy R. Werwie, Hariata G. Tai Rakena, Andrea Medley, Monica M. Desjardins, Lalaine Sevillano, Gwen Wilson, Melissa E. Lewis

**Affiliations:** 1 Center for Indigenous Health, Department of International Health, Johns Hopkins Bloomberg School of Public Health, Johns Hopkins University, 415 N. Washington St., 4th floor, Baltimore, MD 21231 USA; 2 Portland State University, 1825 SW Broadway, Portland, OR 97201 USA; 3 Laureate Institute for Brain Research, Tulsa, OK USA; 4 Oxley School of Community Medicine, University of Tulsa, Tulsa, OK USA; 5 Independent Research Scientist, St. Louis, MO 63124 USA; 6 School of Social Work, University of Michigan, 1080 S University Ave, Ann Arbor, MI 48109 USA; 7 Department of Social Work, California State University Long Beach, 1250 Bellflower Boulevard, Long Beach, CA 90840 USA; 8 Department of Family and Community Medicine, University of Missouri, Columbia, MO USA

**Keywords:** Indigenous, American Indian, Culture, Equity, Protective Factors

## Abstract

Indigenous Peoples experience severe health inequities rooted in experiences of colonization. Calls for strengths-based, culturally grounded research and practice to redress these harms continue to grow. This systematic review assesses how Indigenous “culture” has been quantified in research across the globe over a 10-year span. Following PRISMA guidelines, we searched four databases (2013–2023) for peer-reviewed, English-language articles featuring quantitative measures of Indigenous culture as a positive/protective factor. Data were extracted via a double coding system to assess cultural domains, operationalization strategies, and sociodemographic characteristics of reviewed studies. We reviewed 279 records and identified 461 measures, 289 of which were unique. The most frequently coded cultural domains were connectivity/belonging (47%) and traditional medicine/spirituality (47%), followed by cultural identity (43%) and Indigenous language (36%). Measures most often operationalized culture through behaviors (61%) and beliefs/values (41%). Only 56% of studies reported any psychometric evaluation. Most studies centered on North American contexts and human health outcomes. Efforts to measure Indigenous culture are increasing but remain fragmented, lacking consensus in domain definitions and operational approaches. Results suggest a need for resources to appropriately grapple with and build momentum around ethically and logistically complicated issues surrounding Indigenous cultural measures.

**Supplementary Information:**

The online version contains supplementary material available at 10.1007/s11121-026-01903-5.

## Introduction

Indigenous communities hold cultural practices and values that promote holistic wellbeing (O’Keefe et al., 2022; Ullrich, 2019). Yet across settler colonial nation states, Indigenous Peoples experience among the worst health and socio-economic conditions of any group. For example, American Indian and Alaska Native Peoples have the highest rates of premature mortality, a trend projected to worsen through 2030 (Arias et al., 2022; Bor et al., 2025). Similar patterns are mirrored globally in many Indigenous communities wherein indicators of stress and distress are heightened and fueled in part by displacement and disconnection from homelands and cultural ways (Anderson et al., 2016). Indigenous health inequities are an outcome of colonization (King et al., 2009) and its byproducts including systematic attacks on culture and disproportionate exposure to social drivers of illness like poverty, inadequate infrastructure, reduced access to education, and experiences of discrimination. Thus, unequal health burdens endured by Indigenous Peoples represent a deep injustice.

Redressing these harms must follow Indigenous community priorities where wellbeing is tied to reclaiming pre-colonial cultural values and practices (Bassett et al., 2012; Gone, 2013). Indigenous efforts to revitalize and/or support cultural ways are widespread and growing Jara & Phan (2024); Walker et al., 2024). Slowly, research funds and policy approaches are following suit. For example, grant opportunities and reviewer guidance at U.S. federal and private foundations include emphasis on Indigenous leadership, culturally grounded solutions, and strength-based approaches inclusive of cultural protective factors (Rasmus et al., 2020; RWJF, 2025; Walters et al., 2019). Internationally, Indigenous advocacy has yielded critical shifts in research ethics guidance, funding streams, and reparation work centering Indigenous Peoples. For instance, the Truth and Reconciliation Commission (TRC) of Canada is mandated to share truths with all Canadians about abuses and losses of Indigenous Peoples in residential schools and to promote 94 calls to action for repair. Māori advocacy in Aotearoa (New Zealand) led to establishment of Waitangi Tribunal which enables tribal entities to make claims for reparations and stimulated a shift towards reconciliation nationally. In research spaces, Indigenous principles of “ownership, control, access, and possession” (OCAP®) were created by the First Nations and Inuit Regional Health Survey’s National Steering Committee in 1998. Development and utilization of Kaupapa Māori methodology calls for research led by Indigenous investigators, incorporates Māori knowledge and values, and requires that research benefits Māori communities. This approach necessitates a re-centering of research partnerships to uplift the tino rangatiratanga (sovereignty, self-determination) of Māori.

In alignment with these advancements, research centering Indigenous culture as a protective factor (i.e., protective factors are conditions that protect against harmful or undesirable outcomes *or* enhance/promote positive or desirable outcomes; (Hawkins et al., 2009; Henson et al., 2017)) is promising yet early in development. For example, there is some empirical evidence of “culture” as protective for health, but these findings are not consistent across studies or measures (Walls et al., 2016; Whalen et al., 2022). One reason for substantial gaps in scientific evidence to support Indigenous wisdom lies within the complexity of cultural measurement approaches. First, conceptions of “culture” for all humans tap myriad domains represented by a range of indicators: culture is notoriously complicated in meaning and definition. Kroeber and Kluckhorn’s (1952) classic and widely cited appraisal of human culture identified 164 unique definitions of the term. These generally referenced beliefs, behaviors, values, customs, and language shared within an identity group yet reflected variability across scholars and settings. Relatedly, while *culture* “informs all human behavior,” it remains woefully undefined and understudied in health research (Kagawa-Singer et al., 2016). Relatedly, racial/ethnic identity scholars have long lamented the lack of attention in measurement to multicultural identities and the sociocultural contexts within which they develop (Okazaki & Sue, 2016; Trimble, 2005).

Understanding *Indigenous culture* in particular is indeed complicated by important contextual factors, including (a) specific referencing of a return to pre-colonial ways of being and knowing (Gone, 2007), (b) awareness and implications of being Indigenous in a modern world of multicultural influence and intersecting identities (Trimble, 2005), and (c) attention to the evolution and continuance of Indigenous Peoples to adapt and resist in the face of colonization (Vizenor, 1999). These considerations converge to create “(post)colonial predicaments” (most notably discussed in the context of mismatched mental health needs and services (Gone, 2021)), including diverse perspectives on what may or may not be considered Indigenous culture today. Defining (and measure) Indigenous culture requires consideration of Indigeneity itself. As Lopez and Lucero assert: “The deceptively simple question “Who is indigenous” is fraught with complexity” ((Lopez & Lucero, 2020) p. 294). They describe varying approaches to defining Indigenous group membership, including self-identification and socially constructed notions of “blood quantum” (i.e., imposed conceptions of percentage of American Indian blood). Among other perspectives, the inclusion of individuals as Indigenous can be based on community or tribal claiming, as well as on Indigenous community and cultural connections.

This conceptual mosaic translates to ambiguous approaches to measurement. A concrete illustration lies in findings from a recent scoping review of Indigenous identity (one component of culture) and wellbeing. Study authors found 21 studies employing a quantitative measure of “identity.” Across these, 12 distinct tools were used, each assessing identity in unique ways (Carson et al., 2024). Zooming out, earlier efforts to measure Indigenous culture broadly drew in part upon acculturative stress and/or bicultural identity concepts (Lester, 1999; Oetting et al., 1998a, b). More recent are movements towards strength-based approaches (O’Keefe et al., 2023) that engrain Indigenous ways of knowing, sovereignty, and self-determination in research. These (and other) strategies uniquely touch critical aspects of Indigeneity and underscore the breadth and depth of potential indicators of Indigenous culture.

We have seen these complexities reflected in our own work. We are a group of 12 Indigenous (Native American, First Nations, Māori, Filipino, Ilokano) and three allied scholars with decades of experience doing research with Indigenous communities. Our prior attempts to measure Indigenous culture revealed ambiguity in assessment of cultural indicators, suggesting a need for a comprehensive profiling of existing measures. Unshared understandings of broad domains and dimensions inclusive of vastly diverse Indigenous cultures undermine potential to support community-prioritized culture-based solutions and challenge our ability to ascertain connections between culture and wellbeing. As such, the major research questions guiding this systematic review are (1) What domains of Indigenous “culture” have been assessed by quantitative measures in the published literature globally within 10 years of our initial search? and (2) How are these domains of culture operationalized? We also share sample and setting characteristics and outcomes of focus for all studies reviewed.

## Methods

### Search Strategy

This systematic review follows the Preferred Reporting Items for Systematic Reviews and Meta-Analyses (PRISMA) statement. All records were managed, screened, and extracted in Covidence. A medical librarian (GW) worked with the primary investigators (ML, MW, DB, VO) to develop a search strategy. Four bibliographic databases (PubMed, Cumulative Index to Nursing and Allied Health Literature (CINAHL), PsycINFO with PsycARTICLES, and Scopus) were searched in July 2022 with an updated search in June 2023. Medical Subject Headings (MeSH) included were “Health Services, Indigenous,” “Alaska Natives,” “Inuit,” “Indians, North American,” “Indians, Central American,” “Indians, South American,” “Psychological Tests,” “Psychometrics,” and “Surveys and Questionnaires.” Some additional search terms included indigenous, native, “First Nation,” Aboriginal, enculturation, sociocultural, “cultural continuity,” “traditional foods,” “cultural identity,” “cultural participation,” “cultural connection.” The search was limited to journal articles published 10 years prior to June 2023 (i.e., 2013–2023) and English language.

### Inclusion and Exclusion Criteria

Inclusion criteria were at least 51% of the sample Indigenous, included an “instrument” (i.e., quantitative assessments/measures like survey measures, rating tools, etc.), and contained a measure of Indigenous “culture,” framed as a positive or protective factor (i.e., excluding culturally relevant risks like historical trauma or discrimination). Exclusion criteria included duplicate studies, reviews, commentaries, and editorials.

### Abstract and Full Text Screening

Title and abstract screening were completed by two authors per record and excluded studies tagged to indicate rationale for exclusion. Senior authors (Walls, O'Keefe, Brown, White, and Lewis) were responsible for resolving disagreements. Full text review followed the same procedures. We met biweekly throughout screening stages to discuss unclear records and collectively consider conflicting decisions that could not be resolved by a third author.

### Data Extraction

We began extraction with a virtual meeting during which two authors (Walls & Lewis) provided instruction on extraction goals and steps. Initial extraction templates were tested by the lead author, who created an extraction protocol guide for authors to consult. After a pilot extraction (i.e., working through one article each, deliberating unclear procedures), earlier and mid-career authors were assigned “extractor” roles, and senior authors assigned “checkers” to verify extracted records. Checkers compiled a written summary of any disagreements, suggested changes, and/or verified consensus via an email to individual data extractors. Disagreements that could not be resolved within extractor-checker dyads were brought to biweekly meetings for group discussion.

Given broad definitions of “culture,” we deliberated data extraction processes for cataloging measures for several months. We drew on personal experiences as Indigenous Peoples, professional experiences leading Indigenous health research, prior awareness of multiculturalism frameworks (Georgetown University, 2025), common approaches to measurement development, and a scan of ~ 200 articles appearing in an initial search to create a taxonomy for a two-step measurement extraction approach: (1) domains and (2) operationalization.

*Domains* refer to the concept(s) of focus for a given cultural measure using the following categories:

1. Language (Indigenous language)
2. Food (Indigenous foods with positive spiritual, social, nutritional, or medicinal value)
3. Traditional medicine (e.g., ceremony, using traditional medicines, healing activities, and spirituality)
4. Traditional arts (e.g., weaving, beading, textiles, other crafts)
5. Indigenous identity (positive ethnic identity: proud of my culture, proud of who I am, feeling part of a group, etc.)
6. Cultural knowledge, worldview, values (Indigenous ways of knowing; worldview measures; broad values)
7. Connectivity/connection
8. Continuity (cultural continuity, active collective effort to rehabilitate cultural continuity within communities, often a community level action to revitalize/reclaim culture)
9. Efficacy (ability/sense of belief in ability to overcome that is rooted in culture)
10. Cultural health and wellness (e.g., ascertaining the cultural health of a community)
11. Traditional cultural sports/activities (e.g., lacrosse, stick games)
12. Broad or unspecified (generic, unclear, or covering a broad and/or unspecific range of concepts)
13. Other (write in)
14. Unclear/unable to determine

*Operationalization* involves the process of moving concepts to written tools for assessment. We used the term “operationalization” here to delineate domains of measurement described above from concrete assessment techniques. For example, a measure of “cultural identity” (domain) might be *operationalized* in some studies by way of self-reported beliefs and in others by assessing behaviors. Similarly to our approach to categorizing domains, we aimed to capture a range of operationalization typologies from our own prior experience and review of 200 initial publications:

1. Knowledge (e.g., “Do you know how to…?” Do you speak…?” and “Do you have skills to…?”)
2. Actions/skills/behaviors (e.g., “How often do you…?” Do you participate in…?” and “At what level do you…?”)
3. Beliefs/values/attitudes (“How important are (ABC) to you?” and “Do you believe in…?”
4. Satisfaction: “How satisfied are you…?”
5. Affinity (“How closely do you identify with…?” “How much do you feel close to…?” etc.)
6. Other (write in)
7. Unclear/unable to determine

We extracted several other data points, including sociodemographic data (e.g., participant age, gender, location, and Indigenous groups included) where available. We assessed the general focus/outcome for each record (human health, cultural revitalization/programming, education, arts, or other). For studies focused on human health, we extracted the type of health condition(s) studied (mental health, substance use, suicide, nutrition, aging/Alzheimer’s/dementia, diabetes, infectious disease, cardiovascular disease, cancer, oral health, other). Last, we extracted indication of *any* psychometric assessment of measures (e.g., reliability, validity, and factor structure), coding these as “yes” or “no.”

## Results

**Fig. 1:**
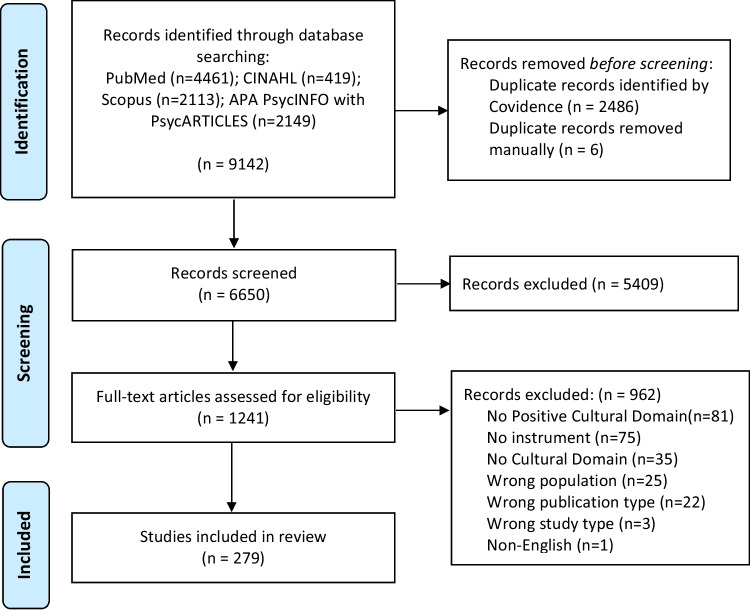
PRISMA flow diagram for record identification, selection, and extraction

The PRISMA diagram for this review appears in Fig. 1. We extracted data for up to five unique measures (scales, indices, etc.) per record. Across 279 records, we assessed 461 measures (not necessarily unique). Reviewers manually entered names for measures as described by publication authors. Using these open-ended responses, we found that 289 of the 461 measures were “unique” (i.e., 172 (37.3%) measures were coded as used in more than one publication). The average number of measures per publication was 1.65 (SD = 1.02); a majority (*n* = 168) of publications had one measure, and* n* = 10 articles included five or more measures. Refer to supplementary online Table for a listing of all measures extracted in this review.

Table 1 displays sociodemographic information across records. American Indians and/or Alaska Natives were the most frequently represented group (44% of all publications), followed by First Nations, Métis, and/or Inuit groups (24%). Native Hawai’ian, Māori, and Aboriginal and/or Torres Strait Islanders were other commonly represented groups. Countries/regions represented in studies reveal further North American focus. Most (81%) records included male and female gender representation; 10% of studies also included non-binary/Two-Spirit participants. Few studies focused solely on early life (e.g., *n* = 7 records centered early to late childhood exclusively) or Elders (*n* = 13 focused on participants aged 50 + years exclusively). The range of publications per year^1^ was 14 (January–June 2023) to 35 (2022; not shown).

**Table 1:** Study characteristics for extracted records

	*n*	%
Indigenous groups represented		
Aboriginal and/or Torres Strait Islander	25	9%
Alaska Native only	14	5%
American Indian only	89	32%
American Indian/Alaska Native	20	7%
First Nations, Métis, and/or Inuit	67	24%
Māori	19	7%
Native Hawaiian	11	4%
Other (Africa, South America, Taiwan, Lebanon, India, etc.)	20	7%
Unspecified, broad, or general Indigenous	14	5%
Countries or regions represented		
Africa	2	1%
Asia/Southeast Asia	5	2%
Australia	26	9%
Canada	74	27%
Mexico	1	0%
Middle East	2	1%
New Zealand	19	7%
North America (US/Can/Mex combination)	5	2%
South America	9	3%
USA	136	49%
Genders represented		
Male, female, Two Spirit and/or non-binary	25	9%
Female, Two Spirit, and/or non-binary	3	1%
Male and female	227	81%
Male only	3	1%
Female only	8	3%
Insufficient data to assess	13	5%
Age groups represented		
0–12 yrs	7	3%
0–17 yrs	24	9%
0–25 yrs	18	6%
0–49 yrs	3	1%
0–50 + yrs	3	1%
13–17 yrs	10	4%
13–25 yrs	17	6%
13–49 yrs	9	3%
13–50 + yrs	17	6%
18–25 yrs	6	2%
18–49 yrs	18	6%
18–50 + yrs	94	34%
26–49 yrs	9	3%
26–50 + yrs	8	3%
50 + yrs	13	5%
Insufficient data to assess	23	8%
Study settings represented		
School	66	16%
Reservation	65	16%
Other	63	16%
Rural	58	14%
Insufficient data to assess	53	13%
Urban	53	13%
Clinic/healthcare setting	28	7%
Social service setting	18	4%

During data extraction, reviewers were able to select multiple categories per demographic characteristic (e.g., multiple age ranges, multiple genders). The disaggregation of age and gender, therefore, does not follow standard groupings, but instead represents the overlapping but distinct representations from a given paper, as cited by reviewers. For example, a subset of papers may include adolescent participants (13–17 yrs), while another included adolescents and young adults (13–25 yrs), while yet another included adolescents, young adults, and older adults (13–49 yrs)—there is overlap, but they are distinct groupings. With regards to study settings, each included article could be coded with multiple settings, including urban or rural in addition to clinic, school, and reservation. Therefore, the total count of study settings exceeds the number of included studies because of the multiple-choice option

We extracted cultural domains of measures in two ways: (1) study authors’ labels (not shown) and (2) our interpretations vis-à-vis measurement descriptions/items. Each measure could be coded with multiple domains. Table 2 displays the percentage of domains represented across measures based on our assessment of measurement descriptions. Two domains tied for highest representation: (1) connectivity/belonging/relationality and (2) traditional medicine/healing/spirituality (each in ~ 47% of records). Cultural identity (43.2%), knowledge/worldviews/values (42.1%), and Indigenous language (36.0%) followed in rank. In* n* = 68 studies (24.5%), coders selected “other” as a domain of measurement and wrote in a brief descriptor. Examples included access to culture, knowledge of history, respect for Elders, resilience, etc. Overall, 7.6% of articles included a cultural domain we were unable to categorize. Across records, reviewers cited an average of 4.3 domains per record/publication and 2.6 domains per measure. Eight measures were coded as representing a single domain; of these eight, a total of two categories were coded as single domains more than once (food *n* = 2; language *n* = 2; data not shown).

**Table 2:** Frequency of domains represented across measures

	*n*	%
Domain		
Connectivity and belonging/family/relationality	132	47.5%
Traditional medicine/healing methods/spirituality	130	46.8%
Identity	120	43.2%
Cultural knowledge, worldview and values	117	42.1%
Language	100	36.0%
Foods (harvest, prepare, eat)	85	30.6%
Broad or unspecified	74	26.6%
Traditional arts, textiles, and activities	68	24.5%
Other	68	24.5%
Traditional cultural sports/activities (e.g., lacrosse, stick ball)	35	12.6%
Cultural health and wellness	28	10.1%
Efficacy	23	8.3%
Unable to determine/unclear	21	7.6%
Continuity	16	5.8%

We assessed study authors’ *operationalization* of cultural domains for each measure (Table 3); more than one code per measure was allowed. Indicators of actions/behaviors were coded most frequently (60.7% of included measures), followed by beliefs/values (41.4%). Studies coded “other” (7.2%) included direct observations or more complex operational definitions cross-cutting multiple concepts (e.g., affinity of friend group + language spoken + language used in thoughts; frequency + importance rankings). In these cases, coders were instructed to choose domain categories as relevant and write in “other” descriptions. A total of *n* = 64 (14%) of records included measures where domain operationalization was unclear.

**Table 3:** Frequency of operationalization approaches across measures

	*n*	%
Operationalization code		
Actions/skills/activities/behaviors	280	60.7%
Beliefs/values	191	41.4%
Knowledge	146	31.7%
Affinity	129	28.0%
Unable to determine/unclear	64	13.9%
Other	33	7.2%
Satisfaction	27	5.9%

We assessed records for indication of *any* form of measurement evaluation or psychometric testing. Slightly over half (56%) included some evaluation of measures, 39% did not, and roughly 5% provided unclear information. Human health was the primary focus in most records (67%; *n* = 228; not shown). Cultural revitalization (9%), education (4%), and the arts (1%) followed as primary foci, and roughly 19% included some “other” focal area (e.g., environmental indicators, policy, religiosity, development, or areas proximal to our broad extraction categories such as cultural health or movement/activity).

## Discussion

This systematic review includes categorization of measures of Indigenous culture appearing in published literature across the globe over a decade of scholarship. We extracted data from 279 records/publications. This existing volume of work including assessment of Indigenous cultural assets is likely a response to community calls to action for strengths-based research and widespread assertion of Indigenous cultural factors as critical to wellbeing (Bassett et al., 2012; O’Keefe et al., 2023).

Cultural connectivity and traditional medicine/spirituality were the most frequently measured domains. Measurement operationalization trended towards tangible actions or behaviors, a potentially sound strategy: in one study we assessed, Fox and colleagues (2018) cite existing evidence that concrete behaviors may be more stable indicators of culture than cognitive or emotional appraisals, particularly during adolescence. Future work should explore age-based distinctions to support developmentally appropriate methods.

Most studies we reviewed centered on North American Indigenous Peoples, rural or reservation/reserve settings, and human health outcomes. This narrow focus limits our understanding of how Indigenous culture could or might best be measured across contexts, offering an incomplete foundation for research inclusive of the vast diversity of Indigenous cultures globally. As one example, the preponderance of reviewed studies assessing Indigenous culture took place in settler colonial nations—places where colonial rule is imposed indefinitely as colonizers seek to take and control lands. How Indigenous cultures persist and evolve in these spaces may “look” differently than in other colonial contexts, including those wherein Indigenous Peoples are exploited for extractive labor (i.e., extractive colonialism) (Shoemaker, 2015).

Overall, we found a lack of clarity in defining and measuring Indigenous culture that limits the impact of empirical evidence to serve as an assessment of its protective aspects. Any given domain of culture (e.g., identity, language, food) was represented across a range of measurement sources and operationalization attempts. For instance, numerous studies assessed cultural identity, defined by us as positive Indigenous ethnic or cultural identity, feeling of pride in culture, or belonging in the cultural group. Multiple records measured identity with multi-domain scales like the Native American Acculturation Scale (NASS) (Garrett & Pichette, 2000), which includes identity-focused items like “how do you identify yourself,” yet also incorporates other aspects of culture like language and friendship. Others utilized measures representing identity as a sole domain. For example, the Multi-Group Ethnic Identity Measure (MEIM) taps multiple dimensions of identity like self-identification, ethnic behaviors, and belonging (example items: “I have a clear sense of my ethnic background and what it means for me,” “I am happy that I am a member of the group I belong to,” and “I have a strong sense of belonging to my own ethnic group”).

These identity-focused examples reiterate our concerns regarding the current state of the science for assessing Indigenous cultural strengths. There is vast heterogeneity in operationalizing identity (and other cultural domains). These multiple approaches could signal a strength given the contextual diversity of Indigenous cultures. Yet this array appears to be driven by a lack of *conceptual* clarity underlying measurement approaches, leading to murky and overlapping operational definitions. The above examples illustrate how empirical assessment of cultural domains might crosscut distinctive conceptualization attempts in prior research (e.g., “belonging” in the MEIM could reasonably be conceived as “connection,” a domain we and others have specified as distinct from identity (e.g., Mohatt et al., 2011; Ullrich, 2019)).

These are not new issues, yet they remain underexplored. Decades of prior research and theory point to the need for *precision* in any quantitative attempts to assess the health-promoting components of ethnicity and/or culture (Sue, 1991). The preponderance of existing research on health and culture broadly (i.e., including beyond Indigenous contexts) too frequently simplifies notions of culture to discrete measurements of race or ethnicity (Kagawa-Singer et al., 2016). Relatedly, our findings illuminate the importance of theory in measurement work. Considering again cultural identity as an illustrative domain, classical theories of identity suggest shifts in identity formation over the life course (Marcia, 1966). These ideas translate in measurement terms to the multi-dimensional nature of many “domains” of culture and signal the need for measures matched to developmental context. Theory further guides deeper thinking on the mechanistic linkages between culture and positive outcomes: it may be, for instance, that mediating or moderating effects of cultural domains are theoretically grounded pathways between other variables and specific behaviors/attitudes as opposed to demonstrating direct effects (Bates et al., 1997; Gonzalez et al., 2022). Further, cultural identities can vacillate and are intersectional *within* groups. For example, health behaviors or positive norms driven by one identity affiliation might not match primary *cultural* identity affiliations across individuals within a single community or ethnic group (Oetting et al., 1998a, b).

Such examples highlight the challenges of encapsulating “culture” and its myriad domains (Walls et al., 2017). A deeper look at “other” domains coded by our team further illustrates murkiness *and* potential expansiveness. Concepts like representation of Indigenous Peoples (television, radio, social media, etc.) and cultural responsibilities for country/land may well relate to broad domains such as identity or connectedness, yet they did not cleanly map onto our pre-defined coding schema. Importantly, we do not view our own attempts to define domains and operationalization of cultural constructs as a benchmark; indeed, we found measurement assessment subjective and complex, even with pre-determined definitions and multiple reviewers. Relatedly, our *team members’ assessment* of cultural measurement domains fully aligned with *authors’ descriptions* in only 16% of the measures we extracted (data not shown). This is likely a conservative estimate given we employed a comprehensive approach to coding: discrepancies in team assessment vs. prior authors’ descriptions are likely not representative of true mutually exclusive conceptions of cultural domains.

This review yields many new questions. Findings indicate largely multi-domain, possibly multi-dimensional attempts to assess Indigenous culture with single measures. Measurement issues aside—including our finding that roughly half of records reviewed explicitly referenced psychometric assessment—questions of whether and/or how Indigenous cultural experiences might be quantified is an ongoing conceptual and ethical concern. Regarding the former, most conceptions of Indigenous culture and wellbeing center holistic perspectives (O’Keefe et al., 2023; Priest et al., 2012; Ullrich, 2019) which are operationally difficult to assess quantitatively. One strategy may be carefully delineating single domains of culture through novel measurement approaches and assessing multiple domains through uniquely specified scales or indices (e.g., multi-measure approaches). This might also enable research partners to understand the nuances of cultural factors most relevant to a given outcome. Humility moving forward in this space is critical: as Robin Wall Kimmerer asks, “Does science allow us to perceive the sacred in the world, or does it bend light in such a way as to obscure it?”.

From an ethical standpoint, Indigenous communities have endured decades of extractive and stigmatizing research injustices (Smith, 2012). Coupling this context of deserved mistrust with centuries of attempted genocide and epistemicide illuminates the depths of care, trust building, and repair necessary in culturally focused research with Indigenous communities. Internationally, Indigenous Peoples lead innovations in community and tribally based participatory research orientations. These approaches encourage community/researcher partnerships, correction of power imbalances in research, and affirm tribal and data sovereignty, including rights of Indigenous Peoples to promote health and cultural resources, oversee research, ensure research is of benefit and relevance to the community, and control access and ownership to data within their territories (Carroll et al., 2020; Harding et al., 2012; Wallerstein & Duran, 2010). Relational approaches are a necessary conduit to addressing community priorities for culture as healing.

Other tensions emerge when considering the relative strengths and weaknesses of generalizable vs. community-specific aspects of cultural measurement, topics also grappled with across prevention science generally (Beals et al., 2003; Stuart et al., 2015). On one hand, researchers and communities might be interested in somewhat generalized measures of culture wherein common indicators can be shared across contexts. For example, asking participants to respond to a question like, “how connected do you feel to your Indigenous culture^2^” might be answerable in most Indigenous spaces. On the other hand, there may be value and richness lost when broad measures miss local or culturally/tribally-specific concepts, practices, or languages. Ultimately, where research teams land on a continuum of specific to generalized measures should be led by community priorities (Walls et al., 2017).

There may also be questionable ethics surrounding assessment of cultural engagement among Indigenous Peoples whose connections were severed due to colonization. For example, we have witnessed that checklist assessments of cultural activities may elicit keen awareness of and distress regarding disconnection and cultural loss. Exploration of diverse Indigenous lifeways and modern expressions of cultural strengths is also needed. For instance, limited empirical attention has been paid to the evolution and continuance of Indigenous thriving and survivance (Vizenor, 1999) despite important discourse on these topics in Indigenous studies and related disciplines (Doerfler et al., 2013; Wilbur & Gone, 2023).

This review includes notable limitations beyond the complexity of conceptualizing and operationalizing culture. We relied exclusively on information available on published records, which may omit key details due to Journal space constraints and variability in measurement reporting standards. Our team is biased by disproportionate North American Indigenous representation; we lack localized expertise important for assessing measures in cross-cultural Indigenous contexts. And, while we attempted to enhance reliability by assigning at least two reviewers per record, interpretations were shaped by individual perspectives and experiences. We provide information regarding the focal areas of research studies extracted, yet we did not assess how or what types of measures are common across fields (e.g., are substance use researchers using similar measures more frequently than, say, chronic disease teams?). We hope that this initial review sparks additional inquiry to highlight research areas that might provide strong models for assessing the protective role of Indigenous culture moving forward.

This study presents initial steps towards assessing and progressing Indigenous cultural measurement approaches. Much more work is needed. It is time for Indigenous scholars and community members to collaboratively create a consensus statement on cultural measurement that emphasizes a “flexibility imperative” (Walters et al., 2019) reflecting equitable approaches and balancing needs for local validity with benefits of generalizable measures (Walls et al., 2017). Funders and reviewers should consider that the state of this science, relative to community calls for stronger culturally grounded, Indigenous-centered approaches is underdeveloped. More resources are needed to fill this gap. Funding to support measurement development and evaluation is uncommon yet critical for Indigenous-focused research. As this work develops, meta-analyses and deeper investigations of measurement approaches will aid understanding of the utility of diverse attempts to measure Indigenous cultural strengths. Overall, these findings underscore a key tension: the richness of Indigenous culture may resist uncluttered quantification. It is our collective responsibility to meet this complexity with rigorous, mixed-methods approaches that honor Indigenous worldviews. Inaction means that measures will obscure more than they reveal and disservice Indigenous community healing.

## Supplementary Information

Below is the link to the electronic supplementary material.

Supplementary file1 (PDF 625 KB)

## Data Availability

Extraction data for this sysematic review are available by request to the authors.

## Footnotes

1 Six records with publication dates between 2010 and 2012 showed discrepancies in Covidence data exports, which listed them as 2023 publications.

2 We note that our use of this potential indicator for illustrative purposes simultaneously unveils many of the complexities discussed in this study. Is this indicator assessing connectedness? Is it a measure of identity or affinity? How might a participant interpret the words “Indigenous” or “culture?”.
