# Supplementary material for: Measuring Indigenous Cultural Strengths: a Systematic Review of a Decade of Approaches
Source: Prev Sci. 2026 Mar 27;27(3):359–68. doi: 10.1007/s11121-026-01903-5 (PMC13102897; doi:10.1007/s11121-026-01903-5)
Supplement: Supplementary file 1 — Supplementary file1 (PDF 625 KB) [file 11121_2026_1903_MOESM1_ESM.pdf]

Author(s)	Year	Title	Measure name	Domains
**Yellowbird**	2011	Examination of the relationship between American Indian cultural identity and academic performance of nursing graduates	Northern Plains Biculturalism Inventory-Revised (NPBI-R)	Language
				Traditional Medicine/Healing Methods/Spirituality
				Identity
				Cultural knowledge, worldview and values
				Connectivity and Belonging/Family/Relationality
				Continuity
				other
**Yasui, Dishion, Stormshak, & Ball**	2015	Socialization of culture and coping with discrimination among American Indian families: Examining cultural correlates of youth outcomes	Youth Ethnic Identity	Identity
				Connectivity and Belonging/Family/Relationality
			Observational Measure Of Parental Cultural Socialization	Cultural knowledge, worldview and values
				Unable to determine/unclear

**Whitesell, Asdigian, Kaufman, Big Crow, Shangreau, Keane, Mousseau, and Mitchell**	2014	Trajectories of substance use among young American Indian adolescents: Patterns and predictors	Cultural Identity	Language
				Traditional arts, textiles, and activities
				Connectivity and Belonging/Family/Relationality
**Urbaeva, Booth, & Wei**	2017	The relationship between cultural identification, family socialization and adolescent alcohol use among Native American families	Native American Cultural Identification	Traditional Medicine/Healing Methods/Spirituality
				Identity
				Cultural knowledge, worldview and values
				Connectivity and Belonging/Family/Relationality
**Tucker, Wingate, O'Keefe**	2015	Historical loss thinking and symptoms of depression are influenced by ethnic experience in American Indian college students	Scale of Ethnic Experience	Identity
				Broad or Unspecified
				other

**Troxela, Palimaru, Kleinb, Dong, Dickersonc et al.**	2022	Changes in sleep-wake patterns and disturbances before and during covid-19 in urban american indian/ alaska native adolescents	Traditional Practices	Traditional arts, textiles, and activities
				Cultural knowledge, worldview and values
				Broad or Unspecified
**Tingey, Rosenstock, Chambers, Patel, Melgar, Slimp et. al.**	2022	Empowering our people: Predictors of retention in an STI risk reduction program among rural Native Americans with binge substance use	Participation in Traditional Activities	Traditional Medicine/Healing Methods/Spirituality
			Participation in Traditional Activities	Traditional arts, textiles, and activities
			Participation In Religious Practices And Ceremonies	Traditional Medicine/Healing Methods/Spirituality
**Linda D. Theis**	2014	Enculturation and posttraumatic growth factors in native americans	American Indian Enculturation Scale	Language
				Foods (Harvest, prepare, eat)
				Traditional Medicine/Healing Methods/Spirituality
				Traditional arts, textiles, and activities
				Identity

				Cultural knowledge, worldview and values
				Connectivity and Belonging/Family/Relationality
			American Indian Enculturation Scale	Language
				Foods (Harvest, prepare, eat)
				Traditional Medicine/Healing Methods/Spirituality
				Identity
				Connectivity and Belonging/Family/Relationality
				Traditional cultural sports/activities (e.g., lacrosse, stick ball)
**Jing Sun & Nicholas Buys**	2013	Using participative community singing program to improve health behaviours in Australian Aboriginal and Torres Strait Islander people	Singing Activity Participation Questionnaire	Traditional arts, textiles, and activities
**Higheagle Strong**	2015	American Indian and Alaska Native self-concept in math and reading: Academic support, ethnic identity, and gender differences	AI/AN Identity	Traditional arts, textiles, and activities
				Broad or Unspecified
**Spillane, Schick, Nalven, Goldstein, Kirk-Provencher et al**	2021	Testing the competing life reinforcers model for substance use in reserve-dwelling First Nation youth	Cultural Reinforcers	Broad or Unspecified

**Shepherd, Delgado, Sivasubramanian, and Paradies**	2018	Predictors of distress and the protective impact of cultural engagement for indigenous prisoners	Cultural Engagement	Cultural knowledge, worldview and values
				Connectivity and Belonging/Family/Relationality
				other
**Carla Santana**	2014	Trails of tears: Implications for removing american indian children from tribal homes	American Indian Enculturation Scale	Language
				Foods (Harvest, prepare, eat)
				Traditional Medicine/Healing Methods/Spirituality
				Traditional arts, textiles, and activities
				Cultural knowledge, worldview and values
				Connectivity and Belonging/Family/Relationality
				Traditional cultural sports/activities (e.g., lacrosse, stick ball)
**Ross**	2018	American Indian Biculturalism Inventory-Pueblo	American Indian Biculturalism Inventory – Pueblo	Identity
				Cultural knowledge, worldview and values
**WanmdiWi J. Rose**	2016	Testing the effect of microaggressions, cultural identity, and gender on learning for Northern Plains American Indian college students	Northern Plains Biculturalism Inventory-Revised (NPBI-R)	Identity
				Cultural knowledge, worldview and values

**Ramsey**	2014	'Aho! all my relations': Native identity and its relationship to psychological well-being	Multi-Group Ethnic Identity Measure (MEIM)	Identity
**Pu, Chewning, St. Clair, Kokotailo, Lacourt et al**	2013	Protective factors in American Indian communities and adolescent violence	Interest In Tribe's Culture	Broad or Unspecified
**Prince, O'Donnell, Stanley, Swaim**	2019	Examination of recreational and spiritual peyote use among American Indian youth	Spiritual Peyote Use	Traditional Medicine/Healing Methods/Spirituality
			Religiosity	Traditional Medicine/Healing Methods/Spirituality
			Orthogonal Cultural Identification Scale	Identity
**Lindsay Price**	2022	Resilience, spirituality and cultural connectiveness within the Native American/American Indian indigenous population	Ethnic, Culture, Religion/Spirituality Scale (Ecrss)	Language
				Traditional Medicine/Healing Methods/Spirituality
				Identity
				Traditional cultural sports/activities (e.g., lacrosse, stick ball)
			Sources of Strenght	Traditional Medicine/Healing Methods/Spirituality
				Connectivity and Belonging/Family/Relationality
			Cultural Connectedness Scale (CCS)	Language
				Foods (Harvest, prepare, eat)
				Traditional Medicine/Healing Methods/Spirituality

				Identity
				Cultural knowledge, worldview and values
				Connectivity and Belonging/Family/Relationality
				Traditional cultural sports/activities (e.g., lacrosse, stick ball)
**Prairie Chicken**	2021	An exploratory factor analysis of the collectivist coping styles inventory using an adult American Indian sample	Collectivist Coping Styles Inventory (Ccs)	Traditional Medicine/Healing Methods/Spirituality
				Connectivity and Belonging/Family/Relationality
			American Indian Biculturalism Inventory - Northern Plains (Aibi-Np)	Identity
**Ponce-Garcia, Calix, Madewell, Randell, Perales, Bread, & Turner**	2019	Through childhood relationship with grandparent, enculturation leads to resilience in Native American adults	American Indian Enculturation Scale	Language
				Traditional Medicine/Healing Methods/Spirituality
				Traditional arts, textiles, and activities
				Traditional cultural sports/activities (e.g., lacrosse, stick ball)
				Broad or Unspecified
			Closeness To Grandparents Scale	Connectivity and Belonging/Family/Relationality
**Phillips**	2021	American Indian and Alaskan native trauma: A program evaluation	Cultural And Traditional Activity Assessment	Traditional Medicine/Healing Methods/Spirituality

			Cultural And Traditional Activity Assessment	Traditional arts, textiles, and activities
			Cultural And Traditional Activity Assessment	Broad or Unspecified
			Cultural Identity	Broad or Unspecified
			Language	Language
			Traditional Activities	Traditional arts, textiles, and activities
				Identity
				Broad or Unspecified
**Patchell**	2011	Native American Indian adolescents: Response to a culturally tailored, school - based substance abuse intervention	Native-Reliance Questionnaire	Language
				Identity
				Connectivity and Belonging/Family/Relationality
				Efficacy
				Broad or Unspecified
**RANDI D. NOEL**	2014	Exploring wellness among American Indian adults	Multi-Group Ethnic Identity Measure (MEIM)	Identity
				Cultural knowledge, worldview and values
				Connectivity and Belonging/Family/Relationality
			American Indian Enculturation Scale	Language
				Foods (Harvest, prepare, eat)
				Traditional Medicine/Healing Methods/Spirituality
				Connectivity and Belonging/Family/Relationality

				Traditional cultural sports/activities (e.g., lacrosse, stick ball)
**Nakoa**	2020	The influence of self-efficacy and Hawaiian identity on academic performance among first year native Hawaiian community college students	Multi-Group Ethnic Identity Measure (MEIM)	Foods (Harvest, prepare, eat)
				Identity
				Cultural knowledge, worldview and values
				Connectivity and Belonging/Family/Relationality
				Broad or Unspecified
			Hawaiian Cultural Identity Scale	Language
				Broad or Unspecified
**Minogue, Delbaere, Radford, Broe, Forder et al.**	2018	Development and initial validation of the Retrospective Indigenous Childhood Enrichment scale (RICE)	Retrospective Indigenous Childhood Enrichment (RICE)	Traditional Medicine/Healing Methods/Spirituality
				Traditional arts, textiles, and activities

				Connectivity and Belonging/Family/Relationality
				Traditional cultural sports/activities (e.g., lacrosse, stick ball)
				Broad or Unspecified
**McCubbin, McCubbin, Zhang, Kehl**	2013	Relational well-being: An indigenous perspective and measure	Relational Well-Being (Rwb)	Language
				Traditional arts, textiles, and activities
				Cultural knowledge, worldview and values
				Connectivity and Belonging/Family/Relationality
				Efficacy
				Cultural health and wellness
**Matika, Manuela, Muriwai, Houkamau, Sibley**	2017	Cultural efficacy predicts increased self esteem for Māori: The mediating effect of rumination	Multi-Dimensional Model Of Māori Identity And Cultural Engagement (MMM-ICE)	Efficacy
**Mason, Rentschler, Habecker, Whitbeck**	2023	Social network analysis of diffusion among american indian youth in a culturally adapted, family-focused prevention program	Ethnic/Cultural Identification Scale	Identity
				Connectivity and Belonging/Family/Relationality
				Broad or Unspecified
			Cultural Socialization Scale	Broad or Unspecified
			Cultural Participation	Traditional Medicine/Healing Methods/Spirituality
				Connectivity and Belonging/Family/Relationality

				Broad or Unspecified
**Manzo**	2015	Suicidal ideation, attempts, and associated psychosocial risk and protective factors in American Indian youth and young adults	Spirituality	Traditional Medicine/Healing Methods/Spirituality
			Bicultural Ethnic Identity Scale	Language
				Traditional Medicine/Healing Methods/Spirituality
				Traditional arts, textiles, and activities
				Identity
**Luke, Anderson, Gee, Thorpe, Rowley, et al.**	2013	Suicide ideation and attempt in a community cohort of urban Aboriginal youth: A cross-sectional study	Culture	Cultural knowledge, worldview and values
				Connectivity and Belonging/Family/Relationality
				other
**Liebenberg, Ikeda, and Wood**	2015	'It's just part of my culture': Understanding language and land in the resilience processes of Aboriginal youth	Child And Youth Resilience Measure (CYRM)	Identity
				Broad or Unspecified
			Cultural Engagement	Language
**Lee**	2017	Culture and history matter: Historical trauma and cultural protective factors on alcohol use among Truku tribal people	Engagement Of Traditional Practices	Foods (Harvest, prepare, eat)
				Traditional Medicine/Healing Methods/Spirituality

				Broad or Unspecified
				other
**Larke, Broe, Daylight, Draper, Cumming, et al.**	2020	Patterns and preferences for accessing health and aged care services in older Aboriginal and Torres Strait Islander Australians	Retrospective Indigenous Childhood Enrichment (RICE)	Broad or Unspecified
				other
**LaPlante, Whelshula, Gray, Nelson**	2021	Understanding American Indian youth in residential recovery from substance use disorder: Risk and protective experiences and perceived recovery support	American Indian Enculturation Scale	Language
				Foods (Harvest, prepare, eat)
				Traditional Medicine/Healing Methods/Spirituality
				Traditional arts, textiles, and activities
				Cultural knowledge, worldview and values
				Connectivity and Belonging/Family/Relationality

				Traditional cultural sports/activities (e.g., lacrosse, stick ball)
**Kaufman, Beals, Croy, Jiang, Novins, The AI-SUPERPFP Team**	2013	Multilevel context of depression in two American Indian tribes	Traditional Ways Of Living	Foods (Harvest, prepare, eat)
				Cultural knowledge, worldview and values
				Connectivity and Belonging/Family/Relationality
			Adherence To Cultural Spirituality	Traditional Medicine/Healing Methods/Spirituality
				Cultural knowledge, worldview and values
				Connectivity and Belonging/Family/Relationality
**Ing, Antonio, Ahn, Cassel, Dillard, Kekauoha, and Keawe'aimoku Kaholokula**	2019	An examination of the relationship between discrimination, depression, and hypertension in Native Hawaiians	Hawaiian Cultural Identity Scale	Cultural knowledge, worldview and values
				Connectivity and Belonging/Family/Relationality
				Broad or Unspecified
**Houkamau, Sibley**	2017	Cultural connection predicts perceptions of financial security for Māori	Multi-Dimensional Model Of Māori Identity And Cultural Engagement (MMM-ICE)	Traditional Medicine/Healing Methods/Spirituality
				Identity
				Cultural knowledge, worldview and values
				Efficacy
**Houkamau, Milojev, Greaves, Dell, Sibley, et al.**	2021	Indigenous ethnic identity, in-group warmth, and psychological wellbeing: A longitudinal study of māori	In-Group Warmth	other

			Ethnic Identity Centrality	Identity
**HIRCHAK**	2018	Examining the relationship between age and treatment outcomes among american indian adults participating in a contingency management clinical trial for alcohol use disorders: A mixed methods approach	American Indian Enculturation Scale	Language
				Foods (Harvest, prepare, eat)
				Traditional Medicine/Healing Methods/Spirituality
				Traditional arts, textiles, and activities
				Cultural knowledge, worldview and values
				Connectivity and Belonging/Family/Relationality

				Broad or Unspecified
**Ciara Dawn Hansen**	2018	Risk and resiliency factors in predicting recidivism among native americans on a montana reservation	Cultural Connectedness Scale (CCS)	Language
				Cultural knowledge, worldview and values
				Connectivity and Belonging/Family/Relationality
				Traditional cultural sports/activities (e.g., lacrosse, stick ball)
				other
**Brenna L. Greenfielda, Kamilla L. Vennerb, J. Scott Toniganb, Monika Honeyestewac, Homer Hubbelld, Dorothea Bluehorse**	2018	Low rates of alcohol and tobacco use, strong cultural ties for Native American college students in the southwest	Traditional Spiritual Activities	Traditional Medicine/Healing Methods/Spirituality
			Cultural Questionnaire	Language
				Traditional arts, textiles, and activities
				Connectivity and Belonging/Family/Relationality
**Greenfield**	2015	Discrimination, substance use, and cultural buffers among Native American college students	Traditional Spiritual Activities	Traditional Medicine/Healing Methods/Spirituality

			Actualization Subscale Of The Urban American Indian Identity Attitudes Scale	Traditional Medicine/Healing Methods/Spirituality
				Identity
			Cultural Questionnaire	Language
				Traditional arts, textiles, and activities
				Connectivity and Belonging/Family/Relationality
				Cultural health and wellness
				Broad or Unspecified
				Unable to determine/unclear
**Greaves, Houkamau & Sibley**	2015	Māori identity signatures: A latent profile analysis of the types of Māori identity	Multi-Dimensional Model Of Māori Identity And Cultural Engagement (MMM-ICE)	Traditional Medicine/Healing Methods/Spirituality
				Identity
				Connectivity and Belonging/Family/Relationality
				Traditional cultural sports/activities (e.g., lacrosse, stick ball)
				other

**Gonzalez, Sittner, Ullrich, Walls**	2021	Spiritual connectedness through prayer as a mediator of the relationship between Indigenous language use and positive mental health	Language	Language
			Prayer/Spiritual Connectedness	Traditional Medicine/Healing Methods/Spirituality
**Gfellner**	2016	Ego strengths, racial/ethnic identity, and well-being among North American Indian/First Nations adolescents	Multi-Group Ethnic Identity Measure (MEIM)	Identity
			Multidimensional Measure Of Racial Identity (Mmri)	Identity
**Gelman**	2017	Using latent variable modeling to explore the factors influencing marijuana use trajectories among American Indian youth	Ethnic Identity (Subset Of Meim)	Language
				Traditional arts, textiles, and activities
				Identity
			Family Cultural Engagement	Traditional Medicine/Healing Methods/Spirituality
				Connectivity and Belonging/Family/Relationality
				Cultural health and wellness
			Cultural Engagement	Language
				Traditional arts, textiles, and activities
			Ethnic Pride	Identity

				Connectivity and Belonging/Family/Relationality
			Community Activities	Connectivity and Belonging/Family/Relationality
				Traditional cultural sports/activities (e.g., lacrosse, stick ball)
**Garrett**	2013	Examining the relationship of cultural identity and historical loss with an American Indian sample on the MMPI-2-RF: A preliminary study	Orthogonal Cultural Identification Scale	Identity
				Cultural knowledge, worldview and values
				Connectivity and Belonging/Family/Relationality
**Fryberg et al.**	2013	Cultural mismatch and the education of Aboriginal youths: The interplay of cultural identities and teacher ratings	The Mcgill Youth Study Team (Myst) Culture And Heritage Scale	Identity
			The Biculturalism Involvement Questionnaire (Biq)	Language
				Traditional arts, textiles, and activities
**Fraser, Geoffroy, Chachamovich, and Kirmayer**	2014	Changing rates of suicide ideation and attempts among inuit youth: A gender-based analysis of risk and protective factors	Community Cohesion	Connectivity and Belonging/Family/Relationality
			Traditional Activities	Foods (Harvest, prepare, eat)
			Cultural Pride	Identity

**Fox, Neha, Jose**	2018	Tū Māori Mai: Māori cultural embeddeness improves adaptive coping and wellbeing for Māori adolescents	Maori Cultural Embeddedness Scale	Language
				Connectivity and Belonging/Family/Relationality
				other
**Fox, Johnson, Winter, Jose**	2023	The Māori Cultural Embeddedness Scale (MaCES): Initial evidence of structural validity	Maori Cultural Embeddedness Scale	other
**Fong, Alejandro, Krou, Segovia, Johnston-Ashton**	2019	Ya'at'eeh: Race-reimaged belongingness factors, academic outcomes, and goal pursuits among indigenous community college students	Belongingness Factors	Connectivity and Belonging/Family/Relationality
**Donovan et al.**	2015	Healing of the Canoe: Preliminary results of a culturally grounded intervention to prevent substance abuse and promote tribal identity for Native youth in two Pacific Northwest tribe	Cultural Identification And Participation In Cultural Activities	Language
				Identity
				other
**Dockery**	2020	Inter-generational transmission of Indigenous culture and children's wellbeing: Evidence from Australia	Longitudinal Study Of Indigenous Children	Identity
				Connectivity and Belonging/Family/Relationality
				Traditional cultural sports/activities (e.g., lacrosse, stick ball)
			Traditional Knowledge	Language

				Foods (Harvest, prepare, eat)
				Traditional Medicine/Healing Methods/Spirituality
				Traditional arts, textiles, and activities
				Traditional cultural sports/activities (e.g., lacrosse, stick ball)
**Dickerson, Brown, Klein, Agniel, Johnson, and D'Amico**	2019	Overt perceived discrimination and racial microaggressions and their association with health risk behaviors among a sample of urban American Indian/Alaska Native adolescents		Traditional Medicine/Healing Methods/Spirituality
				Traditional arts, textiles, and activities
				Traditional cultural sports/activities (e.g., lacrosse, stick ball)
				Broad or Unspecified
**Currie**	2013	Social determinants of alcohol, drug and gambling problems among urban aboriginal adults in Canada	Vancouver Index	Foods (Harvest, prepare, eat)
				Traditional Medicine/Healing Methods/Spirituality
				Identity
				Cultural knowledge, worldview and values

				Connectivity and Belonging/Family/Relationality
				Continuity
**crouch**	2021	Initial development and validation of a quality of life instrument for Alaska native adults: The goodness of life for every Alaska native scale	Goodness Of Life For Every Alaska Native Scale	Language
				Foods (Harvest, prepare, eat)
				Traditional Medicine/Healing Methods/Spirituality
				Identity
				Cultural knowledge, worldview and values
				Connectivity and Belonging/Family/Relationality
				Continuity
				Efficacy

				Cultural health and wellness
				Broad or Unspecified
				other
			Alaska Native Wellness Survey	Language
				Foods (Harvest, prepare, eat)
				Traditional Medicine/Healing Methods/Spirituality
				Traditional arts, textiles, and activities
				Cultural knowledge, worldview and values
				Connectivity and Belonging/Family/Relationality

				Broad or Unspecified
				other
**Casanova**	2011	Ethnic identity, acculturation, and perceived discrimination for indigenous Mexican youth: A cross-cultural comparative study of Yucatec Maya adolescents in the US and Mexico	Maya Language	Language
			Multi-Group Ethnic Identity Measure (MEIM)	Traditional arts, textiles, and activities
				Identity
				Cultural knowledge, worldview and values
				Connectivity and Belonging/Family/Relationality
				other
			Short Acculturation Scale For Hispanics-Youth	Language

				Connectivity and Belonging/Family/Relationality
				other
**Buckingham, Uqiilaq Schroeder, Hutchinson**	2023	Knowing Who You Are (Becoming): Effects of a university-based elder-led cultural identity program on Alaska Native students' identity development, cultural strengths, sense of community, and behavioral health	Native Cultural Health Assessment Tool	Cultural knowledge, worldview and values
				Connectivity and Belonging/Family/Relationality
**Brougham & Haar**	2012	Collectivism, cultural identity and employee mental health: A study of New Zealand Māori	Collectivism Used The Six-Item Scale By Clugston Et Al.	Cultural knowledge, worldview and values
				other
**Brockie**	2012	Historical and contemporary factors influencing non-lethal suicidal behavior among reservation-based native American youth	Communal Mastery Sclae	Connectivity and Belonging/Family/Relationality
			Oetting & Beauvais Orthogonal Cultural Identification Scale (Adapted)	Identity
				Cultural knowledge, worldview and values
				Connectivity and Belonging/Family/Relationality
			Participation in Traditional Activities	Language

				Traditional arts, textiles, and activities
				Cultural knowledge, worldview and values
			Traditional Spirituality	Traditional Medicine/Healing Methods/Spirituality
**Brazill, Myers, Myers, Johnson**	2021	Cultural congruity and academic confidence of American Indian graduate students in STEM: Peer interactions, mentor cultural support, and university environment fit	Cultural Congruity Index	Identity
				Cultural knowledge, worldview and values
				Connectivity and Belonging/Family/Relationality
				other
			Mentor's Cultural Support Index	Cultural knowledge, worldview and values
				Connectivity and Belonging/Family/Relationality
**Brass**	2010	Restoring balance: Determinants of health and depressive symptoms in aboriginal people	Language	Language
				Foods (Harvest, prepare, eat)

				Identity
				Connectivity and Belonging/Family/Relationality
			Orthogonal Cultural Identification Scale	Language
				Traditional arts, textiles, and activities
				Cultural knowledge, worldview and values
**Antonio**	2018	An exploration of resilience among Native Hawaiians	Hawaiian Cultural Identity Scale	Cultural knowledge, worldview and values
				Cultural health and wellness
**Andrade-Bekker**	2015	Power of the powwow: American Indian perspectives on ethnic identity and social interest	Brief Demographic Survey	Identity
				Connectivity and Belonging/Family/Relationality
				other
			Multi-Group Ethnic Identity Measure (MEIM)	Identity
				Connectivity and Belonging/Family/Relationality
				other
			Sulliman Scale Of Social Interest (Sssi)	Unable to determine/unclear
**Allen, Mohatt, Fok, Henry, Burkett, People Awakening Team**	2014	A protective factors model for alcohol abuse and suicide prevention among Alaska Native youth	Elluarrluni Piyugngariluni - Learning In The Mind Of Doing Things In A Masterful Way	Connectivity and Belonging/Family/Relationality

			Elluarrluni Piyugngariluni - Learning In The Mind Of Doing Things In A Masterful Way	Efficacy
			Elluarrluni Piyugngariluni - Learning In The Mind Of Doing Things In A Masterful Way	Connectivity and Belonging/Family/Relationality
			Nunamta: ''Our Community''—Community Characteristics	Connectivity and Belonging/Family/Relationality
				other
			Reflective Processes	Connectivity and Belonging/Family/Relationality
				Continuity
				Efficacy
				Cultural health and wellness
			Yuuyaraqegtaar - A Way To Live A Very Good, Beautiful Life: Reasons For Life	Cultural knowledge, worldview and values
				Connectivity and Belonging/Family/Relationality
				Efficacy
**Allen et al.**	2023	Culturally grounded strategies for suicide and alcohol risk prevention delivered by rural Alaska Native communities: A dynamic wait-listed design evaluation of the Qungasvik intervention	Individual Characteristics (Ip)	Unable to determine/unclear

			Family Characteristics	Unable to determine/unclear
			Community Characteristics	Traditional Medicine/Healing Methods/Spirituality
				Unable to determine/unclear
			Reasons For Life	Traditional Medicine/Healing Methods/Spirituality
				Unable to determine/unclear
			Reflective Processes	Unable to determine/unclear
**Adamsen**	2018	The effects of cultural participation on health outcomes among American Indian/Alaska native elders	Cultural Participation	Foods (Harvest, prepare, eat)
				Broad or Unspecified

**Wilson, Quinn, Abbott, Cairney.**	2017	The role of Aboriginal literacy in improving English literacy in remote Aboriginal communities: an empirical systems analysis with the Interplay Wellbeing Framework	School Culture	Language
				Cultural knowledge, worldview and values
				Broad or Unspecified
			Aboriginal Literacy	Language
**Usuba, Russell, Ritchie, Mishibinijima, Wabano et. al**	2019	Evaluating the Outdoor Adventure Leadership Experience (OALE) program using the Aboriginal Children's Health and Well-being Measure (ACHWM©)	Aboriginal Children's Health And Well-Being Measure (ACHWM)	Traditional Medicine/Healing Methods/Spirituality
				Connectivity and Belonging/Family/Relationality
				Broad or Unspecified
				Unable to determine/unclear
**Unger, Sussman, Begay, Moerner, and Soto**	2020	Spirituality, Ethnic Identity, and Substance Use among American Indian/Alaska Native Adolescents in California	Spirituality Scale Of The Spirituality Measure	Traditional Medicine/Healing Methods/Spirituality
			Multi-Group Ethnic Identity Measure (MEIM)	Identity

			Lifetime Use Of Traditional Tobacco	Traditional Medicine/Healing Methods/Spirituality
**Tassell-Matamua, Lindsay, Bennett, Masters-Awatere**	2021	Māori Cultural Identity Linked to Greater Regard for Nature: Attitudes and (Less So) Behavior	Multi-Dimensional Model Of Māori Identity And Cultural Engagement (MMM-ICE)	Language
				Traditional Medicine/Healing Methods/Spirituality
				Identity
				Connectivity and Belonging/Family/Relationality
				Efficacy
**Spence**	2015	Does social context matter? Income inequality, racialized identity, and health among canada's aboriginal peoples using a multilevel approach	Access To Traditional Medicines, Healing, Wellness Practices.	Traditional Medicine/Healing Methods/Spirituality
				Cultural knowledge, worldview and values
			Aboriginal Language Ability	Language
				Cultural knowledge, worldview and values
**Sowerwine, Mucioki, Sarna-Wojcicki, Hillman**	2019	Reframing food security by and for Native American communities: a case study among tribes in the Klamath River basin of Oregon and California	Adapted Household Food Security Module	Foods (Harvest, prepare, eat)

			Acquisition, Exchange, And Consumption Of Native Foods And Foods-Related Knowledge	Foods (Harvest, prepare, eat)
**Schick, Egan, Crawford, Nalven, Goldstein & Spillane**	2022	Cultural identity affiliation and alcohol use and related consequences among American Indian and White adolescents: A latent profile analysis	American Indian Identity Affiliation	Traditional arts, textiles, and activities
				Cultural knowledge, worldview and values
				Connectivity and Belonging/Family/Relationality
				Broad or Unspecified
**Reid, Varona, Fisher, Smith**	2016	Understanding Maori ‘lived’ culture to determine cultural connectedness and wellbeing	Ngai Tahu Views On Māori Culture	Cultural knowledge, worldview and values
				Connectivity and Belonging/Family/Relationality
				Broad or Unspecified
			Ngai Tahu Cultural Practice In The Previous 12 Months	Language
				Foods (Harvest, prepare, eat)
				Traditional Medicine/Healing Methods/Spirituality

				Traditional arts, textiles, and activities
				Identity
				Cultural knowledge, worldview and values
				Connectivity and Belonging/Family/Relationality
				other
**Pearce, Jongbloed, Richardson, Henderson, Pooyak et al**	2015	The Cedar Project: resilience in the face of HIV vulnerability within a cohort study involving young Indigenous people who use drugs in three Canadian cities Health behavior, health promotion and society	Cultural Connectedness Scale (CCS)	Language
				Traditional Medicine/Healing Methods/Spirituality
				Broad or Unspecified
**Otim, Asante, Kelaher, Doran, Anderson**	2015	What constitutes benefit from health care interventions for Indigenous Australians?	Descriptors Of Indigenous Health	Traditional Medicine/Healing Methods/Spirituality
				Cultural health and wellness
**Kulis, Tsethlikai**	2016	Urban American Indian Youth Spirituality and Religion: A Latent Class Analysis	American Indian Spiritual Practices	Foods (Harvest, prepare, eat)

				Traditional Medicine/Healing Methods/Spirituality
				Cultural health and wellness
				Traditional cultural sports/activities (e.g., lacrosse, stick ball)
				Broad or Unspecified
			Belief Systems	Traditional Medicine/Healing Methods/Spirituality
			Multi-Group Ethnic Identity Measure (MEIM)	Connectivity and Belonging/Family/Relationality
				Broad or Unspecified
			Bicultural Ethnic Identity Scale	Broad or Unspecified
			Language	Language
**Kala**	2022	Traditional Ecological Knowledge of Tribal Communities and Sustainability of Nature and Natural Resources in Pachmarhi Biosphere Reserve in India	Traditional Ecological Knowledge And Practices	Foods (Harvest, prepare, eat)
				Traditional Medicine/Healing Methods/Spirituality
				other

**Nehemiah Ikoba & E.T Jolayemi**	2020	Investigation of Factors Contributing to Indigenous Language Decline in Nigeria	Survey On The Level Of Decline Of Some Indigenous Nigerian Languages	Language
**Houkamau, C. A.; Sibley, C. G.**	2015	The Revised Multidimensional Model of Māori Identity and Cultural Engagement (MMM-ICE2)	Multi-Dimensional Model Of Māori Identity And Cultural Engagement (MMM-ICE)	Traditional Medicine/Healing Methods/Spirituality
				Identity
				Efficacy
				other
**Hossain, Lamb**	2019	Cultural Attachment and Wellbeing Among Canada's Indigenous People: A Rural Urban Divide	Cultural Attachment	Language
				Foods (Harvest, prepare, eat)
				Traditional arts, textiles, and activities
				other
**Hahmann, Perri, Masoud, Bombay**	2023	Parent and/or Grandparent Attendance at Residential School and Dimensions of Cultural Identity and Engagement: Associations with Mental Health and Substance Use among First Nations Adults Living off Reserve	Multi-Group Ethnic Identity Measure (MEIM)	Traditional arts, textiles, and activities
				Identity

				Connectivity and Belonging/Family/Relationality
**Jarrod M. Haar & Dave M. Brougham**	2013	An Indigenous Model of Career Satisfaction: Exploring the Role of Workplace Cultural Wellbeing	Workplace Cultural Wellbeing	Connectivity and Belonging/Family/Relationality
**Gutiérrez-Carmona & Urzúa**	2022	Ethnic Identity and Self-Esteem as Mediators of the Effects of Cultural Involvement on the Wellbeing of Indigenous Andean People	Well-Being Scale In Lickan-Antay People (Bla32)	Traditional Medicine/Healing Methods/Spirituality
				Identity
				Cultural knowledge, worldview and values
				Connectivity and Belonging/Family/Relationality
			Lickan-Antay Scale Of Cultural Involvement (Icla10)	Traditional Medicine/Healing Methods/Spirituality
				Traditional cultural sports/activities (e.g., lacrosse, stick ball)
				Broad or Unspecified

			Multi-Group Ethnic Identity Measure (MEIM)	Identity
				Connectivity and Belonging/Family/Relationality
**Lara M. Greaves, Sam Manuela, Emerald Muriwai, Lucy J. Cowie, Cinnamon-Jo Lindsay, Correna M. Matika, Carla A. Houkamau, & Chris G. Sibley**	2017	The multidimensional model of maori identity and cultural engagement: Measurement equivalence across diverse Maori Groups	Multi-Dimensional Model Of Māori Identity And Cultural Engagement (MMM-ICE)	Language
				Traditional Medicine/Healing Methods/Spirituality
				Identity
				Cultural knowledge, worldview and values
				Connectivity and Belonging/Family/Relationality
				Continuity
				Efficacy

**Lara M. Greaves, Carla A. Houkamau, & Chris G. Sibley**	2017	Random intercept exploratory factor analysis of the multidimensional model of Māori identity and cultural engagement	Multi-Dimensional Model Of Māori Identity And Cultural Engagement (MMM-ICE)	Language
				Traditional Medicine/Healing Methods/Spirituality
				Identity
				Cultural knowledge, worldview and values
				Connectivity and Belonging/Family/Relationality
				Continuity
				Efficacy
**Gallardo-Peralta, Sánchez-Moreno, Rodríguez-Rodríguez**	2019	Strangers in Their Own World: Exploring the Relation Between Cultural Practices and the Health of Older Adults in Native Communities in Chile	Cultural Practice-Related Medicine	Traditional Medicine/Healing Methods/Spirituality
			Intergenerational Transmission Of Cultural Practices	Language

				other
**Forrest**	2018	The intergenerational transmission of Australian Indigenous languages: why language maintenance programmes should be family-focused	Language	Language
**Espín-León et al.**	2021	Effect of Migration Flows on the Ethnic Identity of the Indigenous Peoples of the Amazonian Communities	Waorani Identity Instrument	Traditional Medicine/Healing Methods/Spirituality
				Identity
				Connectivity and Belonging/Family/Relationality
				Broad or Unspecified
				Unable to determine/unclear
**Espín-León, Jimeno-Morenilla, Pertegal-Felices, Azorin-Lopez**	2020	Quantification of cultural identity through artificial intelligence: a case study on the Waorani Amazonian ethnicity	Waorani Identity Instrument	Language
				Foods (Harvest, prepare, eat)
				Traditional Medicine/Healing Methods/Spirituality
				Traditional arts, textiles, and activities
				Identity
				Cultural knowledge, worldview and values

				Connectivity and Belonging/Family/Relationality
				Cultural health and wellness
				Traditional cultural sports/activities (e.g., lacrosse, stick ball)
				Broad or Unspecified
**Baron, Riva, Fletcher, Lynch, Lyonnais, Laouan Sidi**	2021	Conceptualisation and Operationalisation of a Holistic Indicator of Health for Older Inuit: Results of a Sequential Mixed-Methods Project	Aboriginal Language Ability	Language
**Lowe, Wagner, Hospital, Leon Morris, Thompson, et al.**	2019	UTILITY OF THE NATIVE-RELIANCE THEORETICAL FRAMEWORK, MODEL, AND QUESTIONNAIRE	Native-Reliance Questionnaire	Cultural knowledge, worldview and values
				Broad or Unspecified
			Native American Ethnic Identity	other
**Fiedeldey-Van Dijk, Rowan, Dell, Mushquash, Hopkins, et al.**	2016	Honoring Indigenous culture-as-intervention: Development and validity of the Native Wellness Assessment	Native Wellness Assessment (NWA)	Language
				Foods (Harvest, prepare, eat)
				Traditional Medicine/Healing Methods/Spirituality
				Traditional arts, textiles, and activities
				Identity

				Cultural knowledge, worldview and values
				Connectivity and Belonging/Family/Relationality
				Continuity
				Efficacy
				Cultural health and wellness
				Traditional cultural sports/activities (e.g., lacrosse, stick ball)
				Broad or Unspecified
				Unable to determine/unclear
				other
**Young , Wabano, Usuba, Pangowish, Trottier et al.**	2015	Validity of the Aboriginal children's health and well-being measure: Aaniish Naa Gegii?	Aboriginal Children's Health And Well-Being Measure (ACHWM)	Unable to determine/unclear
**Young, Wabano, Ritchie, Burke, Pangowaish, et al.**	2015	Assessing children's interpretations of the Aboriginal Children's Health and Well-Being Measure (ACHWM)	Aboriginal Children's Health And Well-Being Measure (ACHWM)	Language
				Cultural knowledge, worldview and values
				Unable to determine/unclear
**Young, Wabano, Burke, Ritchie, Mishibinijima, et al.**	2013	A process for creating the Aboriginal children's health and well-being measure (ACHWM)	Aboriginal Children's Health And Well-Being Measure (ACHWM)	Traditional Medicine/Healing Methods/Spirituality

				Broad or Unspecified
				Unable to determine/unclear
**Young, Craig, Clapham, Banks, and Williamson**	2018	The prevalence and protective factors for resilience in adolescent Aboriginal Australians living in urban areas: a cross-sectional study	Search - The Study Of Environment On Aboriginal Resilience And Child Health Survey	Cultural knowledge, worldview and values
**Wright, Davis, Brinckley, Lovett, Thandrayen, Yap, Sanders, and Banks**	2022	Relationship of Aboriginal family wellbeing to social and cultural determinants, Central Australia: 'Waltja tjutangku nyakunytjaku'	Kanyini	Cultural knowledge, worldview and values
				Connectivity and Belonging/Family/Relationality
				Efficacy
			Tjukurpa	Language
				Cultural knowledge, worldview and values
				other
			Ngura	Cultural knowledge, worldview and values
				other
			Waltja	Language
				Identity

				Cultural knowledge, worldview and values
				Connectivity and Belonging/Family/Relationality
**Wilson, Johnson, Albino, Jiang, Schmiege, and Brega**	2021	Parental Ethnic Identity and Its Influence on Children's Oral Health in American Indian Families	Ethnic Identity	Language
				Identity
**Weatherly, McDonald, Derenne**	2013	Rates of delay and probability discounting of northern plains American Indians discounting Indian and majority culture-specific outcomes	Northern Plains Biculturalism Inventory-Revised (NPBI-R)	Identity
				Connectivity and Belonging/Family/Relationality
**Walls, Whitbeck, Armenta.**	2016	A Cautionary Tale: Examining the Interplay of Culturally Specific Risk and Resilience Factors in Indigenous Communities	Traditional Spiritual Activities	Foods (Harvest, prepare, eat)
				Traditional Medicine/Healing Methods/Spirituality
				Cultural knowledge, worldview and values
				other
**Wabano, McGregor, L.E., Beaudin, McGregor, L. E.; Kristensen-Didur et. al.**	2019	Health profiles of First Nations children living on-reserve in Northern Ontario: a pooled analysis of survey data	Aboriginal Children's Health And Well-Being Measure (ACHWM)	Language
				Traditional Medicine/Healing Methods/Spirituality

**Viscogliosi, Asselin, Trottier, D'Amours, Levasseaur**	2022	Association between intergenerational solidarity involving elders and mental health of Indigenous people living off reserve	Intergenerational Solidarity	Connectivity and Belonging/Family/Relationality
**Tyser, Scott, Readdy, McCrea**	2013	The role of goal representations, cultural identity, and dispositional optimism in the depressive experiences of American Indian youth from a Northern Plains tribe	Orthogonal Cultural Identification Scale	Language
				Traditional Medicine/Healing Methods/Spirituality
				Cultural knowledge, worldview and values
				Broad or Unspecified
				other
**Tuitt, Asdigian, Mousseau, Ivanich, Zacher et. al.**	2022	Measure of Socialization of American Indian Children (MOSAIC): Understanding the roots of ethnic-racial identity	Measure Of Socialization Of American Indian Children (Mosaic)	Language
				Traditional Medicine/Healing Methods/Spirituality
				Traditional arts, textiles, and activities
				Identity
				Connectivity and Belonging/Family/Relationality

**Bryan Tanner, Sara Plain, Tracey George, Julie George, Christopher J. Mushquash, Sharon Bernards, Melody Morton Ninomiya, and Samantha Wells**	2022	Understanding Social Determinants of First Nations Health Using a Four-Domain Model of Health and Wellness Based on the Medicine Wheel: Findings from a Community Survey in One First Nation	Cultural Resilience Factors	Language
				Cultural knowledge, worldview and values
				Traditional cultural sports/activities (e.g., lacrosse, stick ball)
**Sinclair, Pritchard, McElfish**	2019	An intersectional mixed methods approach to Native Hawaiian and Pacific Islander men's health	Nhpi Values	Identity
				Cultural knowledge, worldview and values
**Sinclair, Gonzales, Woosley, Cree, Garza, & Buchwald**	2020	An Intersectional Mixed Methods Approach to Understand American Indian Men's Health	Physical Activity	Connectivity and Belonging/Family/Relationality
				Traditional cultural sports/activities (e.g., lacrosse, stick ball)
			Eating Habits	Foods (Harvest, prepare, eat)
			Values	Cultural knowledge, worldview and values
				Connectivity and Belonging/Family/Relationality
**Shepherd, Ogloff, Shea, Pfeifer, Paradies**	2017	Aboriginal prisoners and cognitive impairment: the impact of dual	Social And Emotional Wellbeing (Sewb)	Traditional Medicine/Healing Methods/Spirituality

		disadvantage on Social and Emotional Wellbeing		Traditional arts, textiles, and activities
				Identity
				Cultural knowledge, worldview and values
				Connectivity and Belonging/Family/Relationality
				Broad or Unspecified
**Sanders, Oppezzo, Skan, Benowitz, Schnellbaecher et al.**	2022	Demographic and cultural correlates of traditional eating among Alaska Native adults at risk for cardiovascular disease	Food Frequency Questionnaire (FFQ)	Foods (Harvest, prepare, eat)
			Language	Language
**Christopher J. Ryan, Scott T. Leatherdale, Martin J. Cooke**	2016	A cross-sectional examination of the correlates of current smoking among off-reserve First Nations and Métis adults: Evidence from the 2012 Aboriginal Peoples Survey	Aboriginal Language Ability	Language
			Exposure Aboriginal Language	Language
			Hunted, Fished, Or Trapped In Past Year	Foods (Harvest, prepare, eat)
			Traditional Arts And Crafts	Traditional arts, textiles, and activities

**Ryan, Cooke, Leatherdale**	2016	Factors associated with heavy drinking among off-reserve First Nations and Métis youth and adults: Evidence from the 2012 Canadian Aboriginal Peoples Survey	Aboriginal Language Ability	Language
			Cultural Outdoor Activities	Foods (Harvest, prepare, eat)
				Traditional arts, textiles, and activities
			Traditional Arts And Crafts	Traditional arts, textiles, and activities
**Laura C. Rosella PhD, Kathy Kornas MSc, Michael E. Green MD MPH, Baiju R. Shah MD PhD, Jennifer D. Walker PhD, Eliot Frymire MA, Carmen Jones BA**	2020	Characterizing risk of type 2 diabetes in First Nations people living in First Nations communities in Ontario: a population-based analysis using cross-sectional survey data		Language
				Foods (Harvest, prepare, eat)
**Gary Robinson, Eunro Lee, Bernard Leckning, Sven Silburn, Tricia Nagel, Richard Midford**	2022	Validity and reliability of resiliency measures trialled for the evaluation of a preventative Resilience-promoting social-emotional curriculum for remote Aboriginal school students	Child And Youth Resilience Measure (CYRM)	Cultural knowledge, worldview and values
**Richards, Chambers, Begay, Jackson, Tingey, et al.**	2021	Diné (Navajo) female perspectives on mother-daughter communication and cultural assets around the transition to womanhood: a cross-sectional survey	Cultural Assets During The Transition To Womanhood	Traditional Medicine/Healing Methods/Spirituality
				Cultural knowledge, worldview and values

				other
**Poliakova, Riva, Fletcher, Desrochers-Couture, Courtemanche**	2022	Sociocultural factors in relation to mental health within the Inuit population of Nunavik	Traditional Activities	Language
				Foods (Harvest, prepare, eat)
				Traditional Medicine/Healing Methods/Spirituality
				Traditional arts, textiles, and activities
				Cultural knowledge, worldview and values
				Traditional cultural sports/activities (e.g., lacrosse, stick ball)
				other
			Community Activities	Foods (Harvest, prepare, eat)
				Traditional Medicine/Healing Methods/Spirituality
				Connectivity and Belonging/Family/Relationality
				Traditional cultural sports/activities (e.g., lacrosse, stick ball)
			Cultural Identity: Centrality & Connectedness	Language

				Identity
				Cultural knowledge, worldview and values
				Connectivity and Belonging/Family/Relationality
				other
**Paul, McQuaid, Hopkins, Perri, Stewart et al.**	2022	Relations between bullying and distress among youth living in First Nations communities: Assessing direct and moderating effects of culture-related variables	Community Belonging	Connectivity and Belonging/Family/Relationality
			Participation in Traditional Activities	Broad or Unspecified
				other
			Importance Of Traditional Cultural Events	Broad or Unspecified
				other
**Patten, Hiratsuka, Nash, Day, Redwood, et al.**	2021	Smoking Patterns Among Urban Alaska Native and American Indian Adults: The Alaska EARTH 10-Year Follow-up Study	Cultural Factors	Language
				Broad or Unspecified

**Masotti, Dennem, Bañuelos, Seneca, Valerio-Leonce, et al.**	2023	The Culture is Prevention Project: measuring cultural connectedness and providing evidence that culture is a social determinant of health for Native Americans	Cultural Connectedness Scale (CCS)	Traditional Medicine/Healing Methods/Spirituality
				Identity
				other
**Luke, Thorpe, Black, Thorpe, Thomas, et al.**	2021	Collaborative Social-Epidemiology: A Co-analysis of the Cultural and Structural Determinants of Health for Aboriginal Youth in Victorian Schools	Aboriginal Module: Collection And Measurement Of Cultural Determinants	Language
				Foods (Harvest, prepare, eat)
				Traditional Medicine/Healing Methods/Spirituality
				Traditional arts, textiles, and activities
				Cultural knowledge, worldview and values
				Connectivity and Belonging/Family/Relationality

				Traditional cultural sports/activities (e.g., lacrosse, stick ball)
				other
**Lee, Roh, Hsieh, Park**	2023	Determinants of Life Satisfaction and Quality of Life Among American Indian Women Cancer Survivors: The Role of Psychosocial Resources	Functional Assessment Of Chronic Illness Therapy, Spiritual Well-Being Scale 12	Traditional Medicine/Healing Methods/Spirituality
**Larson, Stoeckl, Jarvis, Addison, Grainger et al.**	2020	Indigenous Land and Sea Management Programs (ILSMPs) Enhance the Wellbeing of Indigenous Australians	Wellbeing Impact Evaluation	Language
				Foods (Harvest, prepare, eat)
				Traditional Medicine/Healing Methods/Spirituality
				Cultural knowledge, worldview and values
				Connectivity and Belonging/Family/Relationality
				Efficacy
**Lardon, Wolsko, Tricket, Henry, Hopkins**	2016	Assessing health in an Alaska native cultural context: The Yup'ik Wellness Survey	Yup’ik Wellness Measure	Language
				Foods (Harvest, prepare, eat)
				Traditional Medicine/Healing Methods/Spirituality

				Cultural knowledge, worldview and values
				Connectivity and Belonging/Family/Relationality
				Cultural health and wellness
				Traditional cultural sports/activities (e.g., lacrosse, stick ball)
				other
			Cultural Identity	Identity
				other
**Langham, McCalman, Redman-MacLaren, Hunter, Wenitong**	2018	Validation and Factor Analysis of the Child and Youth Resilience Measure for Indigenous Australian Boarding School Students	Child And Youth Resilience Measure (CYRM)	Broad or Unspecified
**Kelley, Lowe, Greywolf, Wimbish-Tompkinds, & Menon**	2022	A Cultural-Based approach to address substance use among urban Native American young adults	Native-Reliance Questionnaire	Language
				Traditional Medicine/Healing Methods/Spirituality
				Identity
				Cultural knowledge, worldview and values
				Connectivity and Belonging/Family/Relationality

**Kelley, Small**	2016	Establishing the reliability and validity of the sources of strength in one American Indian community	Sources of Strengths	Broad or Unspecified
				other
**Kelley, McCoy, Skye, Singer, Rushing, et al.**	2022	Psychometric evaluation of protective measures in Native STAND: A multi-site cross-sectional study of American Indian Alaska Native high school students	Culture	Identity
				Cultural knowledge, worldview and values
				Efficacy
				Broad or Unspecified
**Kant, Vertinsky, Zheng, Smith**	2013	Social, cultural, and land use determinants of the health and well-being of Aboriginal peoples of Canada: a path analysis	Land Use	Unable to determine/unclear
			Six Domains Of Wellbeing (Including Social-Cultural)	Broad or Unspecified
				Unable to determine/unclear
**Kading, Hautala, Palombi, Aronson, Smith, and Walls**	2015	Flourishing: American Indian Positive Mental Health	Traditional Activities	Foods (Harvest, prepare, eat)
				Traditional Medicine/Healing Methods/Spirituality
				Traditional cultural sports/activities (e.g., lacrosse, stick ball)
				Unable to determine/unclear

**Juric, Batal, David, Sharp, Schwartz, Ing, Fediuk, Black, Tikhonov, Man Chan**	2017	A total diet study and probabilistic assessment risk assessment of dietary mercury exposure among First Nations living on-reserve in Ontario, Canada	Traditional Food Frequency Questionnaire (FFQ)	Foods (Harvest, prepare, eat)
**Jubinville, Smylie, Wolfe, Bourgeois, Berry, et al.**	2022	Relationships to land as a determinant of wellness for Indigenous women, two-spirit, trans, and gender diverse people of reproductive age in Toronto, Canada	Relationship To Land	Broad or Unspecified
				Unable to determine/unclear
			Cultural Connectedness Scale (CCS)	Unable to determine/unclear
			Eats Traditional Foods	Foods (Harvest, prepare, eat)
**Johnson, Okamoto, Rosario, and Pokhrel**	2022	Tobacco product use and cultural connectedness among Native Hawaiian/Pacific Islander, Asian American, and Filipino American young adults in Hawai'i	Cultural Connectedness Scale (CCS)	Foods (Harvest, prepare, eat)
				Cultural knowledge, worldview and values
				Connectivity and Belonging/Family/Relationality
**Ironside, Ferguson, Katapally, Johnson, Foulds**	2023	Associations of Cultural Characteristics with Sedentary Behaviour and Screen Time Among Indigenous Adults in Saskatchewan	Cultural Connectedness Scale (CCS)	Traditional Medicine/Healing Methods/Spirituality
				Identity

				other
			Multi-Group Ethnic Identity Measure (MEIM)	Identity
				Cultural knowledge, worldview and values
				Connectivity and Belonging/Family/Relationality
**Herron, Hirchak, Venner, Tofighi, McDonnell & The Honor Study Team**	2022	Cultural Factors and Alcohol Use in American Indian Adults: Results From a Culturally Tailored Contingency Management Intervention	Addiction Severity Index, Native American Version (ASI-NAV)	Broad or Unspecified
			American Indian Enculturation Scale	Cultural knowledge, worldview and values
				Traditional cultural sports/activities (e.g., lacrosse, stick ball)
				other
**Herbert, Stephens**	2015	Alcohol Use and Older Māori in Aotearoa	Māori Cultural Identity	Language
				Identity
				Connectivity and Belonging/Family/Relationality
				Broad or Unspecified
				other
**Gray, Richer, Harper**	2016	Individual- and community-level determinants of Inuit youth mental wellness	Pride In Inuit Identity	Identity

				Unable to determine/unclear
			Activities Relating To The Land	Foods (Harvest, prepare, eat)
				Unable to determine/unclear
			Difficulty Finding Animals To Hunt	Foods (Harvest, prepare, eat)
**Gonzalez, Aronson, Kellar, Walls, Greenfield**	2017	Language as a Facilitator of Cultural Connection	Language	Language
			Traditional Activities	Traditional arts, textiles, and activities
			Spiritual Activity Index	Traditional Medicine/Healing Methods/Spirituality
			Family Ojibwe Culture	Traditional Medicine/Healing Methods/Spirituality
				Cultural knowledge, worldview and values
				Connectivity and Belonging/Family/Relationality
**Goldstein, Schick, Nalven, & Spillane**	2021	Valuing Cultural Activities Moderating the Association Between Alcohol Expectancies and Alcohol Use Among First Nation Adolescents	Competing Life Reinforcers Measure	Traditional Medicine/Healing Methods/Spirituality
				Traditional arts, textiles, and activities
				Cultural knowledge, worldview and values
**Gilchrist, Hyde, Petersen, Douglas, Hayden, Bessarab,**	2023	Validation of the Good Spirit, Good Life quality-of-life tool for older Aboriginal Australians	Good Spirit, Good Life	Traditional Medicine/Healing Methods/Spirituality

**Flicker, LoGiudice, Ratcliffe, Clinch, Taylor, Bradley, Smith**				Cultural knowledge, worldview and values
				Connectivity and Belonging/Family/Relationality
				Cultural health and wellness
				Broad or Unspecified
				other
			Short Form Of The Aboriginal Resilience And Recovery Questionnaire (Arrq-25)	other
**Gee, Hulbert, Kennedy & Paradies**	2023	Cultural determinants and resilience and recovery factors associated with trauma among Aboriginal help-seeking clients from an Aboriginal community-controlled counselling service	Aboriginal Resilience and Recovery Questionnaire (ARRQ)	Traditional Medicine/Healing Methods/Spirituality
				Identity
				Connectivity and Belonging/Family/Relationality
**Julie Gameon, Monica Skewes**	2021	Historical trauma and substance use among American Indian people with current substance use problems	Multi-Group Ethnic Identity Measure (MEIM)	Identity
				Connectivity and Belonging/Family/Relationality
**Galloway, Johnson-Down, Egeland**	2015	Socioeconomic and Cultural Correlates of Diet Quality in the Canadian Arctic:	Region Specific Food Frequency Questionnaires (Traditional Foods)	Foods (Harvest, prepare, eat)

		Results from the 2007-2008 Inuit Health Survey	Inuit Language	Language
			24 Hour Traditional Food Consumption	Foods (Harvest, prepare, eat)
**Gallardo-Peralta, Fernández-Dávila Jara, Tereucán Angulo, and Rodríguez Martín**	2023	Loneliness among Chilean indigenous women: Family, community, and socio-cultural integration as protective factors	Indigenous Cultural Practices	Foods (Harvest, prepare, eat)
				Traditional Medicine/Healing Methods/Spirituality
				Continuity
			Indigenous Cultural Practices	Traditional Medicine/Healing Methods/Spirituality
				Identity
				Cultural knowledge, worldview and values
				Cultural health and wellness
				Foods (Harvest, prepare, eat)
**Laura Fuentes, Hugo Asselin, Annie Claude Bélisle, Oscar Labra**	2019	Impacts of Environmental Changes on Well-Being in Indigenous Communities in Eastern Canada	Time Spent On The Land	Traditional arts, textiles, and activities
			Support From Family And Friends	Connectivity and Belonging/Family/Relationality
			Life In The Community	Connectivity and Belonging/Family/Relationality
**Findlay, Langlois, Kohen**	2013	Hunger among Inuit children in Canada	Child Traditional Food Intake	Foods (Harvest, prepare, eat)
				Traditional arts, textiles, and activities

			Parent Satisfaction With Support Network	Connectivity and Belonging/Family/Relationality
**Fetter and Thompson**	2023	The impact of historical loss on Native American college students' mental health: The protective role of ethnic identity	Multi-Group Ethnic Identity Measure (MEIM)	Identity
				Connectivity and Belonging/Family/Relationality
**Estes, Sittner, Hill, Gonzalez, Handeland**	2023	Community Engagement and Giving Back Among North American Indigenous Youth	Cultural And Community Involvement	Identity
				Cultural knowledge, worldview and values
				Connectivity and Belonging/Family/Relationality
			Traditional Activities	Traditional Medicine/Healing Methods/Spirituality
				Traditional arts, textiles, and activities
**Dellinger, O'Keefe, Poupart, Stevens, Thompson, Meza, Cassidy**	2022	A Culture and Wellness Pilot to Guide Community Engaged Public Health Research in Native American Populations	Wellness Pilot Survey	Connectivity and Belonging/Family/Relationality
				Cultural health and wellness
				Broad or Unspecified
**Elizabeth J. D'Amico a, Daniel L. Dickersonb, Ryan A. Browna, David J. Kleina, Denis Agniela and Carrie Johnsonc**	2021	Unveiling an 'invisible population': health, substance use, sexual behavior, culture, and discrimination among urban American Indian/Alaska Native adolescents in California	Multi-Group Ethnic Identity Measure (MEIM)	Identity
				Cultural knowledge, worldview and values

				Connectivity and Belonging/Family/Relationality
			Cultural Characteristics	Language
				Traditional Medicine/Healing Methods/Spirituality
				Identity
				Cultural knowledge, worldview and values
				Connectivity and Belonging/Family/Relationality
				Cultural health and wellness
**Chai**	2023	Disability and Suicidal Ideation among Indigenous Adults in Canada: Cultural Resources as Contingencies	Cultural Identity	Identity
			Cultural Group Belonging	Connectivity and Belonging/Family/Relationality
			Cultural Engagement	Broad or Unspecified

				other
			Cultural Exploration	Cultural knowledge, worldview and values
**Brockie, Elm, Walls**	2018	Examining protective and buffering associations between sociocultural factors and adverse childhood experiences among American Indian adults with type 2 diabetes: a quantitative, community-based participatory research approach	Traditional Spiritual Activities	Traditional Medicine/Healing Methods/Spirituality
			Awareness Of Connectedness Scale	Connectivity and Belonging/Family/Relationality
**Brockie, Campbell, Dana-Sacco, Farley, Belcher, Kub, Nelson, Ivanich, Yang, Wallen, Wetsit, & Wilcox**	2022	Cultural Protection from Polysubstance Use Among Native American Adolescents and Young Adults	Adapted Oetting & Beauvais Orthogonal Cultural Identification Scale	Identity
				Cultural knowledge, worldview and values
**Bowker, Gee, Huttlinger**	2021	Development of a Culturally Valid Instrument Examining HPV Knowledge and Beliefs of Lakota Women on the Pine Ridge Reservation	Lakota Cultural Influences (Subscale Of Lakota Women And Cervical Cancer Survey)	Language
				Traditional Medicine/Healing Methods/Spirituality
				Cultural knowledge, worldview and values
				Connectivity and Belonging/Family/Relationality

**Bourke, Chapman, Jones, Brinckley, Thurber et al.**	2022	Developing Aboriginal and Torres Strait Islander cultural indicators: an overview from Mayi Kuwayu, the National Study of Aboriginal and Torres Strait Islander Wellbeing	The Mayi Kuwayu Questionnaire	Language
				Foods (Harvest, prepare, eat)
				Traditional Medicine/Healing Methods/Spirituality
				Traditional arts, textiles, and activities
				Identity
				Cultural knowledge, worldview and values
				Connectivity and Belonging/Family/Relationality
				Efficacy
				Cultural health and wellness

**Blair et al.**	2021	Validation of the Brief Perceived Ethnic Discrimination Questionnaire-Community Version in American Indians	Multi-Group Ethnic Identity Measure (MEIM)	Identity
				Connectivity and Belonging/Family/Relationality
				Unable to determine/unclear
**Berry, Crowe, Deane, and Quinlan**	2022	An exploratory study of culture in treatment for Aboriginal Australian men in residential drug and alcohol rehabilitation services	Aboriginal Cultural Engagement Survey (Aces)	Foods (Harvest, prepare, eat)
				Traditional Medicine/Healing Methods/Spirituality
				Traditional arts, textiles, and activities
				Identity
				Cultural knowledge, worldview and values
				Connectivity and Belonging/Family/Relationality
			General Cultural Engagement In Treatment	other

			Engagement In Specific Treatment Activities	other
**Running Bear, Garroutte, Beals, Kaufman, Manson**	2018	Spirituality and mental health status among Northern Plain tribes	Tribal Cultural Spirituality Measure	Traditional Medicine/Healing Methods/Spirituality
**Barraza, Bartgis, Fresno Native Youth Council**	2016	Indigenous youth-developed self-assessment: The Personal Balance Tool	Adapted Youth Personal Balance Tool	Traditional Medicine/Healing Methods/Spirituality
				Cultural knowledge, worldview and values
				Connectivity and Belonging/Family/Relationality
				Traditional cultural sports/activities (e.g., lacrosse, stick ball)
**Barbic, Young, Usuba, Stankiewicz**	2022	Rasch Measurement Theory's contribution to the psychometric properties of a co-created measure of health and wellness for Indigenous children and youth	Aaniish Naa Gegii: The Children's Health And Wellbeing Measure	Language
				Traditional Medicine/Healing Methods/Spirituality
				Cultural knowledge, worldview and values
				Connectivity and Belonging/Family/Relationality
				Cultural health and wellness

**James Allen, Stacy M. Rasmus, Carlotta Ching Ting Fok, Billy Charles2, Joseph Trimble, KyungSook Lee, and the Qungasvik Team**	2021	Strengths-Based Assessment for Suicide Prevention: Reasons for Life as a Protective Factor From Yup'ik Alaska Native Youth Suicide	Yuuyaraqegtaar - A Way To Live A Very Good, Beautiful Life: Reasons For Life	Traditional Medicine/Healing Methods/Spirituality
				Cultural knowledge, worldview and values
				Efficacy
				other
			Elluarrluni Piyugngariluni - Learning In The Mind Of Doing Things In A Masterful Way	Connectivity and Belonging/Family/Relationality
				other
			Elluarrluteng Ilakelriit - Nurturing Family	Connectivity and Belonging/Family/Relationality
			Nunamta/Our Community: Community Resilience (13 Items).	Connectivity and Belonging/Family/Relationality
			Yuuyaraq/Way Of The Human Being: Awareness Of Connected- Ness (11 Items)	Connectivity and Belonging/Family/Relationality

**Smith, Gilchrist, Talyor, Clinch, ...and Bessarab**	2019	Good Spirit, Good Life: A Quality of Life Tool and Framework for Older Aboriginal Peoples	Good Spirit, Good Life	Language
				Foods (Harvest, prepare, eat)
				Traditional Medicine/Healing Methods/Spirituality
				Connectivity and Belonging/Family/Relationality
				Continuity
				Broad or Unspecified
				other
**Nasreen, Brar, R., Brar, S., Maltby, and Wilk**	2018	Are Indigenous Determinants of Health Associated with Self-Reported Health Professional-Diagnosed Anxiety Disorders Among Canadian First Nations Adults?: Findings from the 2012 Aboriginal Peoples Survey	Aboriginal Peoples Survey Questions On Indigenous Traditional Activities	Foods (Harvest, prepare, eat)
				Traditional arts, textiles, and activities
			Language	Language
**Moghaddam, Momper, Fong**	2013	Discrimination and participation in traditional healing for American Indians and Alaska Natives	Participation In Traditional Healing	Traditional Medicine/Healing Methods/Spirituality

**Helm, Hishinuma, Okamoto, Chin, Silva**	2019	The Relationship Between Ethnocultural Identity Measures and Youth Substance Use Among a School-Based Sample: A Focus on Native Hawaiian Youth	Ethnocultural Identity - 1st Set	Language
				Identity
				other
			Ethnocultural Identity - 2nd Set	Identity
				Connectivity and Belonging/Family/Relationality
**Yetter & Foutch**	2013	Investigation of the structural invariance of the Ethnic Identity Scale with Native American youth	Ethnic Identity	Traditional arts, textiles, and activities
				Identity
				Connectivity and Belonging/Family/Relationality
				other
**Williams, Clark, and Lewycka**	2018	The Associations Between Cultural Identity and Mental Health Outcomes for Indigenous Māori Youth in New Zealand	Maori Cultural Identity Scale (Mcis)	Language
				Traditional Medicine/Healing Methods/Spirituality

				Traditional arts, textiles, and activities
				Identity
				Cultural knowledge, worldview and values
				Connectivity and Belonging/Family/Relationality
**Wexler, Damn, Silvius, Mazziotti, & Bamikole**	2015	Protective factors of native youth: findings from a self-report survey in rural Alaska	Participation In And Attitudes Toward Traditional Culture	Foods (Harvest, prepare, eat)
				Traditional arts, textiles, and activities
				Cultural knowledge, worldview and values
				Cultural health and wellness
				Broad or Unspecified
				other
			Community	Connectivity and Belonging/Family/Relationality
				Efficacy
**Wetherill, Williams, Hartwell, Salvatore, Jacob, et al**	2018	Food choice considerations among American Indians living in rural Oklahoma: The THRIVE study	Adapted Food Choice Values Questionnaire	Foods (Harvest, prepare, eat)

**Weber, Marchman, Diop, Fernald**	2018	Validity of caregiver-report measures of language skill for Wolof-learning infants and toddlers living in rural African villages	Vocabulary Inventory	Language
**Walls, Hautala, Cole, Kosobuski, Weiss, Hill, and Williams**	2022	Socio-cultural integration and holistic health among Indigenous young adults	Cultural Identification	Connectivity and Belonging/Family/Relationality
			Awareness Of Connectedness	Connectivity and Belonging/Family/Relationality
				other
**Walch, Loring, Johnson, Tholl, Bersamin.**	2019	Traditional Food Practices, Attitudes, and Beliefs in Urban Alaska Native Women Receiving WIC Assistance	Traditional Food Acquisition, Practices, Attitudes And Belief	Foods (Harvest, prepare, eat)
				Cultural knowledge, worldview and values
				Connectivity and Belonging/Family/Relationality
**Walch, Bersamin**	2020	Traditional food intake is positively associated with diet quality among low-income, urban Alaska Native women	Food Frequency Questionnaire (FFQ)	Foods (Harvest, prepare, eat)
**Venner, Serier, Sarafin, Greenfield, Hirchak et al.**	2020	Culturally tailored evidence-based substance use disorder treatments are efficacious with an American Indian Southwest tribe: an open-label pilot-feasibility randomized controlled trial	Addiction Severity Index, Native American Version (ASI-NAV)	Traditional Medicine/Healing Methods/Spirituality
				Broad or Unspecified
			Native American Spirituality Scale	Traditional Medicine/Healing Methods/Spirituality

**Usera**	2017	The Efficacy of an American Indian Culturally-Based Risk Prevention Program for Upper Elementary School Youth Residing on the Northern Plains Reservations	Respect	Cultural knowledge, worldview and values
				Connectivity and Belonging/Family/Relationality
				other
			Lakota Identity	Language
				Identity
				Cultural knowledge, worldview and values
				Broad or Unspecified
			Self-Esteem	Identity
**Toombs, Lund, Mushquash, A., Mushquash, C.**	2022	Predictors of Land-Based Activity Participation in a National Representative Sample of Indigenous Individuals Living Off-Reserve	Frequency Of Land-Based Activities	Foods (Harvest, prepare, eat)

				Traditional Medicine/Healing Methods/Spirituality
			Cultural Belonging	Connectivity and Belonging/Family/Relationality
**Tangjitman, Wongsawad, Winijchaiyanan, Sukkho, Kamwong, et. al.**	2013	Traditional knowledge on medicinal plant of the Karen in northern Thailand: A comparative study	Cultural Importance Index	Traditional Medicine/Healing Methods/Spirituality
**Mehdi Taghipoorreyneh1 & Ernest C. de Run**	2020	Using Mixed Methods Research as a Tool for Developing an Indigenous Cultural Values Instrument in Malaysia	Malay Value Items	Cultural knowledge, worldview and values
				Traditional cultural sports/activities (e.g., lacrosse, stick ball)
**A. Sy, M. Greaney, C. Nigg, & S. M. Hirose-Wong**	2015	Developing a measure to evaluate a positive youth development program for Native Hawaiians: the Hui Mālama o ke Kai rubrics of Hawaiian values	Understanding/practice of Hawaiian Culture/Values	Cultural knowledge, worldview and values
				Unable to determine/unclear
			Cultural Identity	Identity
				Unable to determine/unclear
			Child's Hawaiian Values	Cultural knowledge, worldview and values

				Unable to determine/unclear
			Laulima: Cooperation Na'auao: Learned Wiwo'ole: Confidence Lokahi: Harmony Mahalo: Gratitude	Unable to determine/unclear
			Understanding/practice of Hawaiian Culture/Values	Unable to determine/unclear
**Spillane, Greenfield, Venner, and Kahler**	2015	Alcohol use among reserve-dwelling adult First Nation members: use, problems, and intention to change drinking behavior	American Indian Enculturation Scale	Language
				Identity
				Cultural knowledge, worldview and values
				Connectivity and Belonging/Family/Relationality
				other
**Spence**	2015	Does Social Context Matter? Income Inequality, Racialized Identity, and Health Among Canada's Aboriginal Peoples Using a Multilevel Approach	Traditional Medicinal Practices	Traditional Medicine/Healing Methods/Spirituality
			Language	Language
**Soto, Baezconde-Garbanati, Schwartz, and Unger**	2015	Stressful life events, ethnic identity, historical trauma, and participation in cultural activities: Associations with	Multi-Group Ethnic Identity Measure (MEIM)	Identity

		smoking behaviors among American Indian adolescents in California	Cultural Activities	Traditional Medicine/Healing Methods/Spirituality
				Traditional arts, textiles, and activities
**Snowshoe, Crooks, Tremblay, Hinson**	2017	Cultural Connectedness and Its Relation to Mental Wellness for First Nations Youth	Cultural Connectedness Scale (CCS)	Traditional Medicine/Healing Methods/Spirituality
				Identity
				Connectivity and Belonging/Family/Relationality
				Cultural health and wellness
**Snowshoe, Crooks, Tremblay, Craig, and Hinson**	2015	Development of a Cultural Connectedness Scale for First Nations youth	Cultural Connectedness Scale (CCS)	Language
				Foods (Harvest, prepare, eat)
				Traditional Medicine/Healing Methods/Spirituality
				Traditional arts, textiles, and activities
				Identity
				Cultural knowledge, worldview and values
				Connectivity and Belonging/Family/Relationality

				Continuity
				Efficacy
				Cultural health and wellness
**Slater, Bruser, Sutherland, Andrew, Warry, et al.**	2022	Wellness in the Face of Frailty Among Older Adults in First Nations Communities	Determinants Of Health	Language
				Foods (Harvest, prepare, eat)
				Traditional Medicine/Healing Methods/Spirituality
				Traditional arts, textiles, and activities
				other
**Simonds , Omidpanah, Buchwald**	2017	Diabetes prevention among American Indians: the role of self-efficacy, risk perception, numeracy and cultural identity	Cultural Identity	Identity
				Broad or Unspecified
**Simonds, Goins, Krantz, Garroutte**	2014	Cultural identity and patient trust among older American Indians	Patient And Provider Cultural Identity	Traditional Medicine/Healing Methods/Spirituality
				Identity
**Sibley, Houkamau**	2013	The multi-dimensional model of Māori identity and cultural engagement: item response theory analysis of scale properties	Multi-Dimensional Model Of Māori Identity And Cultural Engagement (MMM-ICE)	Broad or Unspecified

**Sheey, Roache, Sharma**	2013	Eating habits of a population undergoing a rapid dietary transition: portion sizes of traditional and non-traditional foods and beverages consumed by Inuit adults in Nunavut, Canada	Traditional Food Intake	Foods (Harvest, prepare, eat)
**Sheehy, T., Kolahdooz, F., Schaefer, S. E., Douglas, D. N., Corriveau, A., et al.**	2014	Traditional food patterns are associated with better diet quality and improved dietary adequacy in Aboriginal peoples in the Northwest Territories, Canada	Culturally Appropriate Quantitative Food Frequency Questionnaire (QFFQ)	Foods (Harvest, prepare, eat)
**Sheehy, Kolahdooz, Roache, Sharma**	2015	Traditional food consumption is associated with better diet quality and adequacy among Inuit adults in Nunavut, Canada	Traditional Meats/Poultry/Fish	Foods (Harvest, prepare, eat)
**Howard & Shea**	2019	Cultural revitalization as a restorative process to combat racial and cultural trauma and promote living well	Awareness Of Connectedness Scale	Language
				Traditional Medicine/Healing Methods/Spirituality
				Traditional arts, textiles, and activities
				Identity
				Cultural knowledge, worldview and values
				Connectivity and Belonging/Family/Relationality
				Efficacy
**Schultz, Abbott, Yamaguchi, Cairney**	2019	Australian Indigenous Land Management, Ecological Knowledge and Languages for Conservation	Participation in Traditional Activities	Foods (Harvest, prepare, eat)

				Traditional Medicine/Healing Methods/Spirituality
				Traditional arts, textiles, and activities
				other
			Spoken Indigenous Language	Language
**Ryan, Cooke, Leatherdale, Kirkpatrick, Wilk**	2015	The correlates of current smoking among adult Métis: Evidence from the Aboriginal Peoples Survey and Métis Supplement	Aboriginal-Specific Determinants Of Health	Language
				Traditional Medicine/Healing Methods/Spirituality
				Traditional arts, textiles, and activities
				Cultural knowledge, worldview and values
				Cultural health and wellness
				Broad or Unspecified
**Ryan, Leatherdale, Cooke**	2016	Factors Associated With Current Smoking Among Off-Reserve First Nations and Métis Youth: Results From the 2012 Aboriginal Peoples Survey	Cultural-Specific Factors	Language
				Traditional Medicine/Healing Methods/Spirituality

				Traditional arts, textiles, and activities
				Traditional cultural sports/activities (e.g., lacrosse, stick ball)
**Richmond, STeckley, Neufeld, Kerr, Wilson and Dokis**	2020	First Nations Food Environments: Exploring the Role of Place, Income, and Social Connection	Food Frequency Questionnaire (FFQ)	Foods (Harvest, prepare, eat)
**Reyes-Garcia, Gueze, Luz, Paneque-Galvez, Macia, Orta-Martinez, Pino, Rubio-Campillo**	2013	Evidence of traditional knowledge loss among a contemporary indigenous society	Measure Of Traditional Knowledge	Foods (Harvest, prepare, eat)
				Traditional Medicine/Healing Methods/Spirituality
				Cultural health and wellness
**Reeds, Mansuri, Mamakeesick, Harris, Zinman, et. al.**	2016	Dietary Patterns and Type 2 Diabetes Mellitus in a First Nations Community	Food Frequency Questionnaire (FFQ)	Foods (Harvest, prepare, eat)
**Redwood, Day, Beans, Hiratsuka, Nash, et al**	2019	Alaska Native Traditional Food and Harvesting Activity Patterns over 10 Years of Follow-Up	Earth Diet History Questionnaire (Dhq)	Foods (Harvest, prepare, eat)
**Ratelle, Skinner, Packull-McCormick, Laird**	2020	Food frequency questionnaire assessing traditional food consumption in Dene/Métis communities, Northwest Territories, Canada	Food Frequency Questionnaire (FFQ)	Foods (Harvest, prepare, eat)
				Cultural knowledge, worldview and values
**Rahman, Ullah, Ali, Aziz, Alam, et. al.**	2022	Traditional knowledge of medicinal flora among tribal communities of Buner Pakistan	Cultural Importance Index	Traditional Medicine/Healing Methods/Spirituality
				Broad or Unspecified

**Pearce, Jongbloed, Pooyak, Blair, Sharma, et al.**	2018	The Cedar Project: exploring determinants of psychological distress among young Indigenous people who use drugs in three Canadian cities	Frequency Family Had Lived By Traditional Culture	Cultural knowledge, worldview and values
				Broad or Unspecified
			Frequency Family Had Spoken Traditional Languages At Home	Language
			Language	Language
			Frequency Of Living By Traditional Culture	Broad or Unspecified
			Participation in Traditional Activities	Foods (Harvest, prepare, eat)
				Traditional Medicine/Healing Methods/Spirituality
				Broad or Unspecified
**Patterson, Wolf, Welte, Barnes, Tidwell, et al.**	2015	Sociocultural Influences on Gambling and Alcohol Use Among Native Americans in the United States	Native American-Specific Demographic Factors	Language
				Identity

				Cultural knowledge, worldview and values
				other
			Participation in Traditional Activities	Foods (Harvest, prepare, eat)
				Traditional Medicine/Healing Methods/Spirituality
				Traditional arts, textiles, and activities
				Cultural knowledge, worldview and values
				Traditional cultural sports/activities (e.g., lacrosse, stick ball)
				Broad or Unspecified
				other
			Bicultural Ethnic Identity Scale	Cultural knowledge, worldview and values
				Broad or Unspecified
			Native American Identity Scale	Foods (Harvest, prepare, eat)
				Traditional Medicine/Healing Methods/Spirituality
				Identity

				Cultural knowledge, worldview and values
				Connectivity and Belonging/Family/Relationality
				Broad or Unspecified
			Composite Native American Orientation Measure	Language
				Foods (Harvest, prepare, eat)
				Traditional Medicine/Healing Methods/Spirituality
				Traditional arts, textiles, and activities
				Identity
				Cultural knowledge, worldview and values
				Broad or Unspecified
**Palimaru, Dong, Brown, D'Amico, Dickerson et al.**	2022	Mental health, family functioning, and sleep in cultural context among American Indian/Alaska Native urban youth: A mixed methods analysis	Cultural Identity	Identity
**Oster, Grier, Lightning, Mayan, and Toth**	2014	Cultural continuity, traditional Indigenous language, and diabetes in Alberta First Nations: a mixed methods study	Indigenous Language Knowledge Rates	Language
				Continuity

				other
**Olko, Lubiewska, Maryniak, Haimovich, de la Cruz, et al.**	2022	The positive relationship between Indigenous language use and community-based well-being in four Nahua ethnic groups in Mexico	Language	Language
**Oetzel, Ruru, Zhang, Simpson, Nock et al.**	2021	Enhancing Well-Being and Social Connectedness for Māori Elders Through a Peer Education (Tuakana-Teina) Programme: A Cross-Sectional Baseline Study	Cultural Identity	Unable to determine/unclear
			Understanding Of Tikanga (Cultural Protocols)	Broad or Unspecified
				Unable to determine/unclear
**Oetzel,Hokowhitu, Simpson, Reddy, Cameron, et al.**	2019	Correlates of Health-Related Quality of Life for Māori Elders Involved in a Peer Education Intervention	Cultural Identity	Identity
			Importance Of Whānau/Family	Connectivity and Belonging/Family/Relationality
			Knowledge Of Tikanga	Traditional Medicine/Healing Methods/Spirituality
				Cultural knowledge, worldview and values
				Cultural health and wellness
**O'Brien, Thummel, Bulkow, Wang, Corbin**	2017	Declines in traditional marine food intake and vitamin D levels from the 1960s to present in young Alaska Native women	Traditional Food Consumption	Foods (Harvest, prepare, eat)

**Norden-Krichmar, Gizer, Libiger, Wilhelmsen, Ehlers, Schork**	2017	Correlation analysis of genetic admixture and social identification with body mass index in a Native American community	Orthogonal Cultural Identification Scale	Identity
				Broad or Unspecified
			Indian Culture Scale	Traditional Medicine/Healing Methods/Spirituality
				Traditional arts, textiles, and activities
				Traditional cultural sports/activities (e.g., lacrosse, stick ball)
				Broad or Unspecified
**Sarah Lynn Newell, Michelle L Dion, Nancy C Doubleday**	2019	Cultural continuity and Inuit health in Arctic Canada	Identity	Identity
			Services In Aboriginal Language	Continuity
			Harvesting Activity	Foods (Harvest, prepare, eat)
			Government Satisfaction	other
**Mousseau, Scott, Estes**	2014	Values and depressive symptoms in American Indian youth of the Northern Plains: examining the potential moderating roles of outcome expectancies and perceived community values	Tradition/Benevolence Sub-Scale	Connectivity and Belonging/Family/Relationality
				Broad or Unspecified

				other
**Masotti, Dennem, Hadani, Banuelos, King, et al.**	2020	The Culture is Prevention Project: Measuring Culture As a Social Determinant of Mental Health for Native/Indigenous Peoples	Cultural Connectedness Scale (CCS)	Traditional Medicine/Healing Methods/Spirituality
				Identity
				other
**Marushka, Kenny, Batal, Cheung, Fediuk, et al.**	2019	Potential impacts of climate-related decline of seafood harvest on nutritional status of coastal First Nations in British Columbia, Canada	Traditional Food Frequency Questionnaire (FFQ)	Foods (Harvest, prepare, eat)
**Martin**	2017	Culture and identity: LSIC parents' beliefs and values and raising young indigenous children in the twenty-first century	Indigenous Status	Identity
			Identifies With Mob(S)	Identity
			Importance Of Being Indigenous	Identity
				Cultural knowledge, worldview and values
			Knowledge Of Family, History, Culture	Broad or Unspecified
			Passing On Indigenous Culture To Children	Language
				Foods (Harvest, prepare, eat)
				Traditional Medicine/Healing Methods/Spirituality

				Traditional arts, textiles, and activities
				Identity
				Cultural knowledge, worldview and values
				Connectivity and Belonging/Family/Relationality
				other
**Stephen S. Kulis, Danielle E. Robbins, Tahnee M. Baker, Serena Denetsosie, and Nicholet A. Deschine Parkhurst**	2016	A latent class analysis of urban American Indian youth identities	Markstrom's Model Of Local Identity	Language
				Foods (Harvest, prepare, eat)
				Traditional Medicine/Healing Methods/Spirituality
				Identity
			Multi-Group Ethnic Identity Measure (MEIM)	Foods (Harvest, prepare, eat)
				Identity

				Cultural knowledge, worldview and values
				Connectivity and Belonging/Family/Relationality
				Broad or Unspecified
			Bicultural Ethnic Identity Scale	Foods (Harvest, prepare, eat)
				Traditional Medicine/Healing Methods/Spirituality
				Traditional arts, textiles, and activities
				Cultural knowledge, worldview and values
				Connectivity and Belonging/Family/Relationality
**Kulis, Ayers, Harthun**	2017	Substance Use Prevention for Urban American Indian Youth: A Efficacy Trial of the Culturally Adapted Living in 2 Worlds Program	AI Ethnic Identity	Foods (Harvest, prepare, eat)
				Traditional arts, textiles, and activities

				Identity
				Connectivity and Belonging/Family/Relationality
				Cultural health and wellness
				Broad or Unspecified
			AI Spirituality	Traditional Medicine/Healing Methods/Spirituality
**Kulis, Wagaman, Tso, Brown**	2013	Exploring Indigenous Identities of Urban American Indian Youth of the Southwest	Connection: Component Of Markstrom's Model Of Local Identity	Identity
				Connectivity and Belonging/Family/Relationality
				other
			Spiritual: Cultural/Spiritual Component Of Markstrom's Model Of Local Identity	Traditional Medicine/Healing Methods/Spirituality
				Cultural knowledge, worldview and values
			Language: Cultural/Spiritual Component Of Markstrom's Model Of Local Identity	Language
			Cultural Traditions And Practices: Cultural/Spiritual Of Markstrom's Model Of Local Identity	Foods (Harvest, prepare, eat)
				Traditional Medicine/Healing Methods/Spirituality
				Cultural knowledge, worldview and values

				Connectivity and Belonging/Family/Relationality
				Continuity
				Cultural health and wellness
				Traditional cultural sports/activities (e.g., lacrosse, stick ball)
			Bicultural Ethnic Identity Scale	Identity
				Connectivity and Belonging/Family/Relationality
**Stephen Kulis, Stephanie L. Ayers, and Tahnee Baker**	2015	Parenting in 2 Worlds: pilot results from a culturally adapted parenting program for urban American Indians	Ethnic Identity Commitment And Attachment	Connectivity and Belonging/Family/Relationality
				Broad or Unspecified
			Ethnic Identity Exploration	Traditional Medicine/Healing Methods/Spirituality
				Broad or Unspecified
			American Indian Way Of Life	Cultural knowledge, worldview and values
				Broad or Unspecified
			Spirituality	Traditional Medicine/Healing Methods/Spirituality

**Kolahdooz, Butler, Lupu, Sheehy, Corriveau, Sharma**	2014	Assessment of dietary intake among Inuvialuit in Arctic Canada using a locally developed quantitative food frequency questionnaire	Culturally Appropriate Quantitative Food Frequency Questionnaire (QFFQ)	Foods (Harvest, prepare, eat)
**King, Masotti, Dennem, Hadani, Linton, et al.**	2019	The Culture is Prevention Project: Adapting the Cultural Connectedness Scale for Multi-Tribal Communities	Cultural Connectedness Scale (CCS)	Language
				Foods (Harvest, prepare, eat)
				Traditional Medicine/Healing Methods/Spirituality
				Identity
				Cultural knowledge, worldview and values
				Connectivity and Belonging/Family/Relationality
				Efficacy
			Cultural Connectedness Scale (CCS)	Traditional Medicine/Healing Methods/Spirituality
				Identity
				other
**Kightley, Reyes-García, Demps, Magtanong, Ramenzoni**	2013	An empirical comparison of knowledge and skill in the context of traditional ecological knowledge	Knowledge	Traditional arts, textiles, and activities
			Skill	Traditional arts, textiles, and activities

**Kickett-Tucker, Christensen, Lawrence, Zubrick, Johnson, Stanley**	2015	Development and validation of the Australian Aboriginal racial identity and self-esteem survey for 8-12 year old children (IRISE_C)	Irise_c	Identity
				Cultural knowledge, worldview and values
**Kelley, Lowe**	2018	A Culture-Based Talking Circle Intervention for Native American Youth at Risk for Obesity	Cherokee Self-Reliance	Identity
				Cultural knowledge, worldview and values
				other
**Kelley, Witzel, Fatupaito**	2019	Preventing Substance Use in American Indian Youth: The Case for Social Support and Community Connections	Culture	Cultural knowledge, worldview and values
			Community Connections	Language
				Cultural knowledge, worldview and values
				Connectivity and Belonging/Family/Relationality
**Kelley, Small**	2020	Healers Need Healing Too: Results from the Good Road of Life Training	Sources of Strength	Broad or Unspecified
**Jessie Kai, John J. Chen, Kathryn L. Braun, Joseph Keawe'aimoku Kaholokula, Rachel Novotny, Carol J. Boushey & Marie K. Fialkowski**	2022	Associations between Cultural Identity, Household Membership and Diet Quality among Native Hawaiian, Pacific Islander, and Filipino Infants in Hawai'i	Cultural Identity	Identity
				Cultural knowledge, worldview and values

				Broad or Unspecified
**Kabir, Hasan, Rahman, M., Rahman, A., Khan**	2014	A survey of medicinal plants used by the Deb barma clan of the Tripura tribe of Moulvibazar district, Bangladesh	Traditional Medicinal Practices	Traditional Medicine/Healing Methods/Spirituality
**Merrill Jones & Renee V. Galliher**	2014	Daily racial microaggressions and ethnic identification among Native American young adults	Multi-Group Ethnic Identity Measure (MEIM)	Foods (Harvest, prepare, eat)
				Identity
				Connectivity and Belonging/Family/Relationality
			Orthogonal Cultural Identification Scale	Language
				Traditional Medicine/Healing Methods/Spirituality
				Cultural knowledge, worldview and values
				Connectivity and Belonging/Family/Relationality
**Janssen, Lévesque, and Xu,**	2014	Correlates of physical activity among First Nations children residing in First Nations communities in Canada		Language
				Foods (Harvest, prepare, eat)
				Cultural knowledge, worldview and values
				Connectivity and Belonging/Family/Relationality

				Broad or Unspecified
**Ironside, Ferguson, Katapally, Hedayat, Johnson, Foulds**	2021	Social determinants associated with physical activity among Indigenous adults at the University of Saskatchewan	Traditional Indigenous Physical Activity	Foods (Harvest, prepare, eat)
				Traditional Medicine/Healing Methods/Spirituality
				Traditional cultural sports/activities (e.g., lacrosse, stick ball)
**Ironside, Ferguson, Katapally, Foulds**	2020	Cultural connectedness as a determinant of physical activity among Indigenous adults in Saskatchewan	Cultural Connectedness Scale (CCS)	Cultural knowledge, worldview and values
				Connectivity and Belonging/Family/Relationality
				Broad or Unspecified
			Multi-Group Ethnic Identity Measure (MEIM)	Identity
**Hodge, Stemmler, Nandy**	2014	Association between Obesity and History of Abuse among American Indians in Rural California	Cultural/Social Life	Language
				Traditional Medicine/Healing Methods/Spirituality
				Connectivity and Belonging/Family/Relationality
				Traditional cultural sports/activities (e.g., lacrosse, stick ball)
**Guenzel, N.; Struwe, L.**	2020	Historical Trauma, Ethnic Experience, and Mental Health in a Sample of Urban American Indians	Scale of Ethnic Experience	Identity
**Greenfield, Hallgren, Venner, Hagler, Simmons, et al.**	2015	Cultural adaptation, psychometric properties, and outcomes of the Native American Spirituality Scale	Native American Spirituality Scale	Traditional Medicine/Healing Methods/Spirituality

			Scale of Ethnic Experience	Identity
**Arla Good, Lori Sims, Keith Clarke, Frank A. Russo**	2019	Indigenous youth reconnect with cultural identity: The evaluation of a community- and school-based traditional music program		Cultural knowledge, worldview and values
				Connectivity and Belonging/Family/Relationality
				Broad or Unspecified
**Gonzalez, Sittner, & Walls**	2022	Cultural efficacy as a novel component of understanding linkages between culture and mental health in Indigenous communities	American Indian Enculturation Scale	Language
				Foods (Harvest, prepare, eat)
				Traditional Medicine/Healing Methods/Spirituality
				Cultural knowledge, worldview and values
				Traditional cultural sports/activities (e.g., lacrosse, stick ball)
			Cultural Efficacy	Connectivity and Belonging/Family/Relationality
				Efficacy
**George, MacLeod, Graham, Plain, Bernards, Wells**	2018	Use of Traditional Healing Practices in Two Ontario First Nations	Participation in Traditional Healing	Traditional Medicine/Healing Methods/Spirituality
			First Nation Identity	Identity

				other
**Fidji Gendron, Anna Hancherow, and Ashley Norton**	2017	Exploring and revitalizing Indigenous food networks in Saskatchewan, Canada, as a way to improve food security		Foods (Harvest, prepare, eat)
**Gendron**	2017	Aboriginal youth's perceptions of traditional and commercial tobacco in Canada	Youths' Perceptions Of Traditional And Commercial Tobacco	Traditional Medicine/Healing Methods/Spirituality
				Cultural knowledge, worldview and values
**Eva Garroutte, Heather Anderson, Patricia Nez-Henderson, Calvin Croy, Janette Beals, Jeffery Henderson, Jacob Thomas and Spero Manson**	2014	Religio-Spiritual Participation in Two American Indian Populations	Spiritual Participation	Traditional Medicine/Healing Methods/Spirituality
**Carina Fiedeldey-Van Dijk, Margo Rowan, Colleen Dell, Chris Mushquash, Carol Hopkins, Barb Fornssler, Laura Hall, David Mykota, Marwa Farag & Bev Shea**	2016	Honoring Indigenous culture-as-intervention: Development and validity of the Native Wellness Assessment(TM)	Native Wellness Assessment (NWA)	Language
				Foods (Harvest, prepare, eat)
				Traditional Medicine/Healing Methods/Spirituality
				Traditional arts, textiles, and activities

				Identity
				Cultural knowledge, worldview and values
				Connectivity and Belonging/Family/Relationality
				other
**Ferguson, Brown, Georga, Miles, Wilson, Brimblecombe**	2017	Traditional food availability and consumption in remote Aboriginal communities in the Northern Territory, Australia	Frequency And Variety Of Traditional Food Consumption	Foods (Harvest, prepare, eat)
**Dyall, Kepa, Teh, Mules, Moyes, Wham, Hayman, Connolly, Wilkinson**	2014	Cultural and social factors and quality of life of Maori in advanced age. Te puawaitanga o nga tapuwae kia ora tonu - Life and living in advanced age: a cohort study in New Zealand (LiLACS NZ)	Life And Living In Advanced Age: A Cohort Study In New Zealand (Lilacs Nz) Questionnaire - Cultural Practices Of Māori In Advanced Age	Language
				Identity
				Cultural knowledge, worldview and values

				Connectivity and Belonging/Family/Relationality
				Continuity
				Cultural health and wellness
**Donovan, Thomas, Sigo, Prie, Lonczak, et al.**	2015	Healing of the canoe: preliminary results of a culturally tailored intervention to prevent substance abuse and promote tribal identity for Native youth in two Pacific Northwest tribes	Cultural Identification	Traditional arts, textiles, and activities
				Identity
				Cultural knowledge, worldview and values
				other
**Dill, Manson, Jiang, Pratte, Gutilla, Knepper, Beals, Roubideaux, and Special Diabetes Program for Indian Diabetes Prevention Demonstration Project**	2016	Psychosocial Predictors of Weight Loss among American Indian and Alaska Native Participants in a Diabetes Prevention Translational Project	Spirituality	Traditional Medicine/Healing Methods/Spirituality
				Cultural knowledge, worldview and values

				Connectivity and Belonging/Family/Relationality
**Dickerson, D'Amico, Klein, Johnson, Hale, Ye**	2021	Mental Health, Physical Health, and Cultural Characteristics Among American Indians/Alaska Natives Seeking Substance Use Treatment in an Urban Setting: A Descriptive Study	Addiction Severity Index, Native American Version (ASI-NAV)	Traditional Medicine/Healing Methods/Spirituality
			Additional Traditional Practice Participation	Traditional Medicine/Healing Methods/Spirituality
				Traditional arts, textiles, and activities
			Urban American Indian Identity Attitude Scale	Identity
**D'Amico**	2020	Motivational interviewing and culture for urban Native American youth (MICUNAY): A randomized controlled trial	Intentions To Participate In Traditional Practices	Traditional cultural sports/activities (e.g., lacrosse, stick ball)
			Cultural Pride And Belonging	Identity
			Spirituality	Traditional Medicine/Healing Methods/Spirituality
**Cwik, Rosenstock, Tingey, Redmond, Gokish, Larzelere-Hinton, Barlow**	2017	Exploration of Pathways to Binge Drinking Among American Indian Adolescents	Cultural Identity	Traditional Medicine/Healing Methods/Spirituality
				Identity
				Cultural knowledge, worldview and values

**Currie, Wild, Schopflocher, Laing, Veugelers**	2013	Illicit and prescription drug problems among urban Aboriginal adults in Canada: the role of traditional culture in protection and resilience	Vancouver Index	Unable to determine/unclear
			Unnamed/No Citation	Traditional arts, textiles, and activities
				Cultural knowledge, worldview and values
				Connectivity and Belonging/Family/Relationality
				Broad or Unspecified
**Currie, Copeland, Metz, Moon-Riley, and Davies**	2020	Past-Year Racial Discrimination and Allostatic Load Among Indigenous Adults in Canada: The Role of Cultural Continuity	Cultural Continuity	Continuity
**Currie, Copeland, Metz**	2019	Childhood racial discrimination and adult allostatic load: The role of Indigenous cultural continuity in allostatic resiliency	Cultural Continuity	Identity
				Connectivity and Belonging/Family/Relationality
				other
**Crooks**	2017	Two Years of Relationship-Focused Mentoring for First Nations, Métis, and Inuit Adolescents: Promoting Positive Mental Health	Cultural Connectedness Scale (CCS)	Identity
				Connectivity and Belonging/Family/Relationality

**Rushing, Stephanie Craig ; Kelley, Allyson ; Bull, Sheana ; Stephens, David ; Wrobel, Julia ; Silvasstar, Joshva ; Peterson, Roger ; Begay, Corey ; Dog, Thomas Ghost ; McCray, Celena ; Brown, Danica Love ; Thomas, Morgan ; Caughlan, Colbie ; Singer, Michelle ; Smith, Paige ; Sumbundu, Kanku**	2021	Efficacy of an mHealth Intervention (BRAVE) to Promote Mental Wellness for American Indian and Alaska Native Teenagers and Young Adults: Randomized Controlled Trial	BRAVE Survey	Traditional Medicine/Healing Methods/Spirituality
				Identity
				Cultural knowledge, worldview and values
				Connectivity and Belonging/Family/Relationality
				Efficacy
**Felina M. Cordova-Marks, DrPH, James K. Cunningham, PhD, Robin B. Harris, PhD, Lynn B. Gerald, PhD, Beatrice Norton, Ann M. Mastergeorge, PhD, and Nicolette I. Teufel-Shone, PhD**	2020	Resilience and Stress among Hopi Female Caregivers		Language
				Traditional Medicine/Healing Methods/Spirituality

				Traditional arts, textiles, and activities
			Connor - Davidson Resilience Scale (Cd-Risc-10)	other
**Clarke, Douglas, Campos, House, Vaughn, Hudgins**	2022	Building connection and improving health for Indigenous elders: Findings from the Title VI evaluation	Social Connectedness	Connectivity and Belonging/Family/Relationality
			Cultural Connectedness Scale (CCS)	Foods (Harvest, prepare, eat)
				Traditional arts, textiles, and activities
				Broad or Unspecified
				other
**Clark, Walton, Drolet, Tribute, Jules, Main, Arnouse**	2013	Melq'ilwiye: coming together--intersections of identity, culture, and health for urban Aboriginal youth	Indigenous Identity And Resistance	Language
				Identity
				Continuity
				Broad or Unspecified
				other

			Cultural Connectedness Scale (CCS)	Language
				Traditional Medicine/Healing Methods/Spirituality
				Unable to determine/unclear
			Culturally Safe Health Care	Unable to determine/unclear
				other
**Chee, Shorty, Kurpius**	2019	Academic stress of Native American undergraduates: The role of ethnic identity, cultural congruity, and self-beliefs	Ethnic Identity	Identity
			Cultural Congruity Index	Cultural knowledge, worldview and values
				Connectivity and Belonging/Family/Relationality
**Carlson, Aronson, Unzen, Lewis, Benjamin, Walls**	2017	Apathy and Type 2 Diabetes among American Indians: Exploring the Protective Effects of Traditional Cultural Involvement	Cultural Involvement	Traditional Medicine/Healing Methods/Spirituality
			Cultural Involvement	Foods (Harvest, prepare, eat)
				Traditional arts, textiles, and activities
				Unable to determine/unclear
**Caqueo-Urizar, FLores, Mena-Chamorro, Urzua, Irarrázaval**	2021	Ethnic identity and life satisfaction in indigenous adolescents: The mediating role of resilience	Multi-Group Ethnic Identity Measure (MEIM)	Identity
				Connectivity and Belonging/Family/Relationality

**Campos, Araujo, Gaoue, Albuquerque**	2019	Socioeconomic Factors and Cultural Changes Explain the Knowledge and Use of Ouricuri Palm (Syagrus coronata) by the Fulni–ô Indigenous People of Northeast Brazil		Language
				Traditional arts, textiles, and activities
				Connectivity and Belonging/Family/Relationality
**Cairney, Abbott, Quinn, Yamaguchi, Wilson, Wakerman**	2017	Interplay wellbeing framework: a collaborative methodology 'bringing together stories and numbers' to quantify Aboriginal cultural values in remote Australia	Interplay Survey	Cultural knowledge, worldview and values
				Connectivity and Belonging/Family/Relationality
				Cultural health and wellness
				other
**Burnett, Purkey, Davison, Watson, Kehoe, Traviss, Nolan, Bayoumi**	2022	Spirituality, Community Belonging, and Mental Health Outcomes of Indigenous Peoples during the COVID-19 Pandemic	Sense Of Community Belonging	Connectivity and Belonging/Family/Relationality
			Importance Of Spirituality Before And During The Covid-19 Pandemic	Traditional Medicine/Healing Methods/Spirituality
**Brown, Dickerson, Klein, Agniel, Johnson, D'Amico**	2021	Identifying as American Indian/Alaska Native in Urban Areas: Implications for	AI/AN Traditional Practices	Traditional cultural sports/activities (e.g., lacrosse, stick ball)

		Adolescent Behavioral Health and Well-Being		Broad or Unspecified
			Cultural Pride And Belonging	Identity
				Connectivity and Belonging/Family/Relationality
			Spirituality/Happiness	Traditional Medicine/Healing Methods/Spirituality
**Brega, Henderson, Harper, Thomas, Manson, Batliner, Braun, Quissell, Wilson, Tiwari, Albino**	2019	Association of Ethnic Identity with Oral Health Knowledge, Attitudes, Behavior, and Outcomes on the Navajo Nation	Ethnic Identity	Language
				Identity
				Cultural knowledge, worldview and values
**Blanchet, Willows, Johnson, Okanagan Nation Salmon Reintroduction Initatives, Batal**	2022	Enhancing cultural food security among the Syilx Okanagan adults with the reintroduction of Okanagan sockeye salmon	Traditional Food Frequency Questionnaire (FFQ)	Foods (Harvest, prepare, eat)
			Cultural Food Security	Foods (Harvest, prepare, eat)
**Blanchet, Batal, Johnson-Down, Johnson, Okanagan Nation Salmon Reintroduction Initiatives, & Willows**	2021	An Indigenous food sovereignty initiative is positively associated with well-being and cultural connectedness in a survey of Syilx Okanagan adults in British Columbia, Canada	Cultural Connectedness Scale (CCS)	Foods (Harvest, prepare, eat)
				Traditional Medicine/Healing Methods/Spirituality
				Identity

				Cultural knowledge, worldview and values
				Connectivity and Belonging/Family/Relationality
				Cultural health and wellness
				Traditional cultural sports/activities (e.g., lacrosse, stick ball)
			Traditional Food Frequency Questionnaire (FFQ)	Foods (Harvest, prepare, eat)
			Cultural Food Security	Foods (Harvest, prepare, eat)
**Bersamin, Wolsko, Luick, Boyer, Lardon, Hopkins, Stern, Zidenberg-Cherr**	2014	Enculturation, perceived stress, and physical activity: implications for metabolic risk among the Yup'ik--the Center for Alaska Native Health Research Study	American Indian Enculturation Scale	Broad or Unspecified
**Bersamin, Izumi, Nu, O'Brien, Paschall**	2019	Strengthening adolescents' connection to their traditional food system improves diet quality in remote Alaska Native communities: results from the Neqa Elicarvigmun Pilot Study	Attitudes And Beliefs Around Traditional Foods, Focusing On Salmon	Foods (Harvest, prepare, eat)
			American Indian Enculturation Scale	Cultural knowledge, worldview and values

**Baydoun**	2015	Ethnopharmacological survey of medicinal plants used in traditional medicine by the communities of Mount Hermon, Lebanon	Cultural Importance Index	Traditional Medicine/Healing Methods/Spirituality
**Batal**	2021	Comparison of measures of diet quality using 24-hour recall data of First Nations adults living on reserves in Canada	Traditional Food Consumption	Foods (Harvest, prepare, eat)
**Batal, Man Chan, Fediuk, Ing, Berti, Sadik, Johnson-Down**	2021	Importance of the traditional food systems for First Nations adults living on reserves in Canada	Food Frequency Questionnaire (FFQ)	Foods (Harvest, prepare, eat)
**Justine Ashokar**	2016	Societal participation difficulties and cultural beliefs about family and spiritual ceremonies in Oglala Sioux Native Americans with and without traumatic brain injury	Native American Cultural Values And Belief Survey-Revised (Nacvbs-R) Subscale: Participation In Spiritual Ceremonies	Traditional Medicine/Healing Methods/Spirituality
**Mapuana C. K. Antonio, Earl S. Hishinuma, Claire Townsend Ing, Fumiaki Hamagami, Adrienne Dillard, B. Puni Kekauoha, Cappy Solatorio, Kevin Cassel, Kathryn L. Braun, Joseph Keawe'aimoku Kaholokula**	2020	A Resilience Model of Adult Native Hawaiian Health Utilizing a Newly Multi-Dimensional Scale	Hawaiian Cultural Identity Scale	Identity
				Cultural knowledge, worldview and values
				Connectivity and Belonging/Family/Relationality
				Efficacy
**Antonio, Ann, Ing, Dillard, Cassel, Kekauoha, Kaholokula**	2016	Self-Reported Experiences of Discrimination and Depression in Native Hawaiians	Hawaiian Cultural Identity Scale	Identity
				Unable to determine/unclear

**Adamsen, Manson & Jiang**	2021	The Association of Cultural Participation and Social Engagement With Self-Reported Diagnosis of Memory Problems Among American Indian and Alaska Native Elders	Cultural Participation	Broad or Unspecified
